# A progesterone biosensor derived from microbial screening

**DOI:** 10.1038/s41467-020-14942-5

**Published:** 2020-03-09

**Authors:** Chloé Grazon, R C. Baer, Uroš Kuzmanović, Thuy Nguyen, Mingfu Chen, Marjon Zamani, Margaret Chern, Patricia Aquino, Xiaoman Zhang, Sébastien Lecommandoux, Andy Fan, Mario Cabodi, Catherine Klapperich, Mark W. Grinstaff, Allison M. Dennis, James E. Galagan

**Affiliations:** 1 Department of Chemistry, Boston University, Boston, MA 02215 USA; 2 University Bordeaux, CNRS, Bordeaux INP, LCPO, UMR 5629, F-33600 Pessac, France; 3 Department of Microbiology, Boston University, Boston, MA 02118 USA; 4 National Emerging Infectious Diseases Laboratories, Boston University, Boston, MA 02118 USA; 5 Department of Biomedical Engineering, Boston University, Boston, MA 02215 USA; 6 Division of Materials Science and Engineering, Boston University, Boston, MA 02215 USA

**Keywords:** Genomics, Synthetic biology, Biosensors, Biomedical engineering

## Abstract

Bacteria are an enormous and largely untapped reservoir of biosensing proteins. We describe an approach to identify and isolate bacterial allosteric transcription factors (aTFs) that recognize a target analyte and to develop these TFs into biosensor devices. Our approach utilizes a combination of genomic screens and functional assays to identify and isolate biosensing TFs, and a quantum-dot Förster Resonance Energy Transfer (FRET) strategy for transducing analyte recognition into real-time quantitative measurements. We use this approach to identify a progesterone-sensing bacterial aTF and to develop this TF into an optical sensor for progesterone. The sensor detects progesterone in artificial urine with sufficient sensitivity and specificity for clinical use, while being compatible with an inexpensive and portable electronic reader for point-of-care applications. Our results provide proof-of-concept for a paradigm of microbially-derived biosensors adaptable to inexpensive, real-time sensor devices.

## Abstract (web summary)

Bacteria represent an unexploited reservoir of biosensing proteins. Here the authors use genomic screens and functional assays to isolate a progesterone sensing allosteric transcription factor and use a FRET-based method to develop an optical progesterone sensor.

## Introduction

Biosensors underlie applications ranging from medical diagnostics, environmental monitoring, food and water safety, to the detection of chemical or biological threats^1^. A typical biosensor utilizes a biorecognition element coupled to a transduction mechanism^2,3^. Yet the number and types of biorecognition elements are limited. The gold standard for clinical hormone analysis uses antibodies as the biorecognition element which are expensive to design and manufacture, do not provide an intrinsic read-out, and do not readily support repeated or continuous measurements.

Bacteria have evolved over 3 billion years to detect and respond to a wide range of stimuli^4–13^. One common molecular sensing mechanism is an allosteric transcription factor (aTF). Binding of ligand to aTFs leads to differential binding of the TF to its cognate DNA binding sites and an alteration in gene expression. Allosteric TFs are used as biosensors in whole cell applications^14^, but the use of bacterial cells in sensors is limited by slow response times, biosafety concerns, and the practical limitations of using a cellular host^15^. Moreover, characterized TFs are a small fraction of the hundreds of thousands of known TFs that have been sequenced^10^, and sequenced genes are only a tiny sampling of the diversity of microbes^16^. Here, we report an approach to identify and harvest microbial aTFs specific to a target analyte, and a transduction strategy for engineering these into sensor devices. We show the application of our approach to develop an optical progesterone sensor based on a previously uncharacterized microbial aTF identified with our screening approach.

## Results

### A progesterone sensing aTF

Our screening approach employs a combination of genomic and functional assays for the targeted identification, validation, and characterization of TF biosensors for specific analytes (Supplementary Fig. 1). Our approach (Supplementary Fig. 1) is based on three observations: bacterial TFs commonly bind upstream of their genes to regulate their own promoters or those of adjacent genes^17–21^, genes for the metabolism of analytes are often found in genome clusters^22^, and these clusters are often induced by their substrates via TFs in genomic proximity^23–26^. We applied our approach to identify bacterial aTFs responsive to steroid hormones owing to their central roles in human physiology, wellness, and health. Steroids are also found widely in nature due to contamination by human activity and natural production by plants, fungi, and animals^27^. Consequently a host of environmental bacteria are able to use steroids as carbon and energy sources^28^. One steroid metabolizing bacterium is *Pimelobacter simplex* (also *Nocardioides simplex* and formerly *Corynebacterium simplex*) which biotransforms a range of steroids^29,30^. Though *P. simplex* is employed in industry for the metabolism of steroids^31^, little is known about the corresponding regulatory mechanisms.

**Fig. 1:**
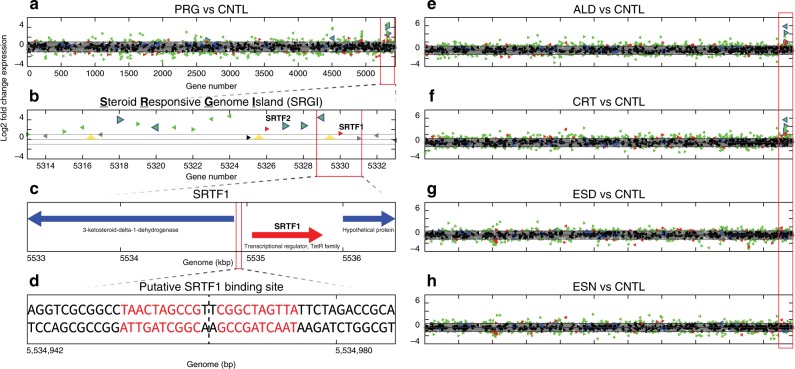
Screening of *P. simplex* for steroid sensing aTFs. **a**–**e** Log2-fold change expression in response to each hormone relative to control. Each triangle is a gene (red = differentially expressed TF, black = non-differentially expressed TF, blue = gene with annotated sterol related function, green = differentially expressed other gene, gray = non-differentially expressed other gene). The Steroid Responsive Gene Island (SRGI) that is differentially expressed in response to PRG, ALD, and CRT is boxed in red. SRTF1 and SRTF2 are two steroid responsive TFs. **f** Zoomed in view of SRGI. Yellow peaks are experimentally validated binding sites for SRTF1. **g** SRGI region around SRTF1. **h** SRTF1 palindromic binding site upstream of SRTF1 gene. RNA-seq data are deposited in the Gene Expression Omnibus with accession number GSE141603.

*P. simplex* was screened to identify aTFs capable of sensing steroid hormones. Cultures of *P. simplex* were exposed to aldosterone, cortisol, estrone, estradiol, progesterone and control media (Supplementary Fig. 2). RNA Sequencing (RNA-Seq) was used to identify gene clusters and associated TFs induced by steroids (Fig. 1). One such gene cluster was significantly upregulated on exposure to progesterone, aldosterone, and cortisol and was termed the Steroid Responsive Genomic Island (SRGI). (Fig. 1a–e, Supplementary Table 1). No differential expression of this cluster was observed for estrone or estradiol (Supplementary Table 1). The SRGI overlaps a previously reported cluster of genes experimentally associated with steroid metabolism^29,32^ and includes one annotated 3-ketosteroid-9-α-hydroxylase, two annotated 3-ketosteroid-δ1-dehydrogenases, and one annotated steroid δ-isomerase (Supplementary Table 1).

Importantly, the SRGI includes three predicted but functionally uncharacterized TF genes^29^ (Fig. 1f, Supplementary Table 1): (1) a predicted TetR-family TF in a divergent promoter with a predicted ketosteroid-dehydrogenase gene, (2) a predicted IclR-family TF adjacent to a second ketosteroid-dehydrogenase gene, and (3) a predicted MarR-family TF upstream of the IclR-TF. Only the first two TFs (whose proteins were termed SRTF1 and SRTF2, respectively) display induction by sterols in our experiments (Fig. 1f). Analysis of the divergent intergenic region upstream of the SRTF1 gene revealed a 22 bp palindromic sequence immediately upstream of the adjacent ketosteroid-dehydrogenase gene (Fig. 1h). We confirmed binding to this palindrome by SRTF1 using a method for in vitro chromatin-immunoprecipitation followed by sequencing (in vitro ChIP-Seq). In vitro ChIP-Seq discovered two additional binding sites in the cluster including one upstream of SRTF2 (Supplementary Fig. 3). These data suggest that SRTF1 mediates the steroid responsiveness of both ketosteroid-dehydrogenase genes and SRTF2. We thus focused additional analysis on SRTF1.

**Fig. 2:**
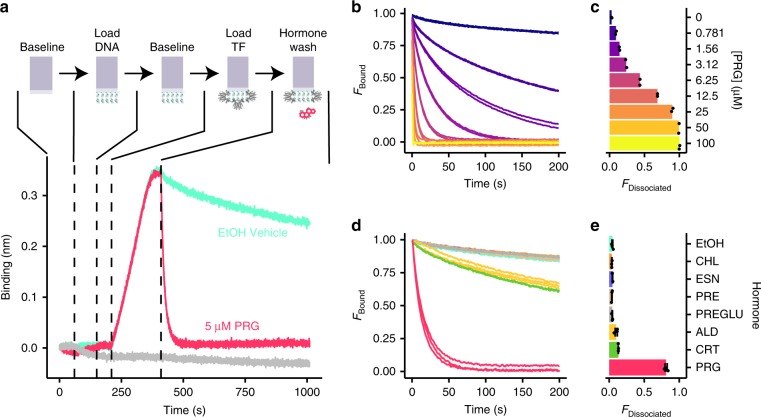
SRTF confirmed as a progesterone responsive aTF. **a** Top panel: BioLayer Interferometry (BLI) experimental approach. Bottom panel, binding layer thickness on probe normalized to baseline. Probes were loaded with oligos containing the SRTF1 binding site (red line) or a scrambled sequence (gray line). Addition of progesterone resulted in rapid unbinding of SRTF1 (red line) that was not observed with vehicle control (cyan line). **b** Unbinding of SRTF1 is dose-responsive. Dissociation curves in duplicate were normalized such that binding at time 0 was equal to 1. **c** Fraction SRTF1:DNA complex from curves shown in panel **b** dissociated at 5 s. **d** Dissociation curves in triplicate of SRTF1 in the presence of 5 μM various steroids showing strongest induction by progesterone normalized as in panel **b**. **e** Fraction SRTF1:DNA complex from curves shown in panel **d** dissociated at 30 s. Error bars are standard error over three experiments. Data underlying bar graphs in panels **c** and **e** are available in the Source Data file.

We quantitatively confirmed that SRTF1 is a sterol responsive aTF using biolayer interferometry (BLI)^33^ (Fig. 2). Synthetic oligonucleotides containing either the predicted palindromic site found upstream of SRTF1 or a shuffled control sequence were conjugated to the BLI probe (Fig. 2a). The probe was placed in a solution with purified SRTF1 protein and no steroids. SRTF1 rapidly bound to the oligo containing the predicted binding sequence, while no binding was observed to the scrambled sequence, further confirming the specificity of the predicted binding motif (Fig. 2a). The probe was then moved into a solution with no protein and either vehicle or 5 μM steroid hormones (Fig. 2a, b, d). Minimal unbinding was observed with vehicle, and a quantitative analysis indicated nanomolar affinity of SRTF1 to the oligonucleotide in the absence of sterols (Supplementary Fig. 8, Supplementary Table 2). In contrast, SRTF1 rapidly dissociated after exposure to progesterone, with 81% dissociation at 30 s after exposure compared to 4.5% in vehicle (Fig. 2d, e). This change in DNA binding was highly specific to progesterone. Exposure to cholesterol or the estrogens beta-estradiol or estrone resulted in no SRTF1 dissociation relative to control, while exposure to aldosterone or cortisol yielded only 10 and 13% dissociation, respectively (Fig. 2d, e). Moreover, no differences in dissociation were observed after exposure to 5β-Pregnane-3α,20-α-diol (pregnanediol) and 5β-Pregnane-3α,20-α-diol-glucuronide (pregnanediol-glucuronide) (Fig. 2d, e). Both are urine metabolites of progesterone^34^, and antibodies against pregnanediol-glucuronide are the basis of many tests for the indirect measurement of progesterone^35^. Experiments with varying concentrations of progesterone demonstrated that progesterone-induced dissociation was dose-dependent (Fig. 2b, c). Together, these data confirm that SRTF1 is an aTF that binds its cognate DNA site in the absence of sterol hormones and allosterically rapidly unbinds in the presence of progesterone. To our knowledge, this is the first example of a progesterone-sensing bacterial transcription factor.

### A QD-TF-FRET framework for aTF biosensors

Using aTFs as an affinity-based biorecognition element in a biosensor requires converting analyte binding into a detectable signal. Antibodies are the benchmark for affinity-based biosensors^3^. However, antibodies bound to an analyte are inert. Detection of binding typically requires the addition of secondary antibodies, and this additional step prevents real-time and continuous measurements and prohibits multiple measurements^3^. In contrast, aTFs combine affinity recognition with a built-in mechanism for the real-time transduction of reversible analyte binding: the reversible binding to their cognate DNA binding sites.

**Fig. 3:**
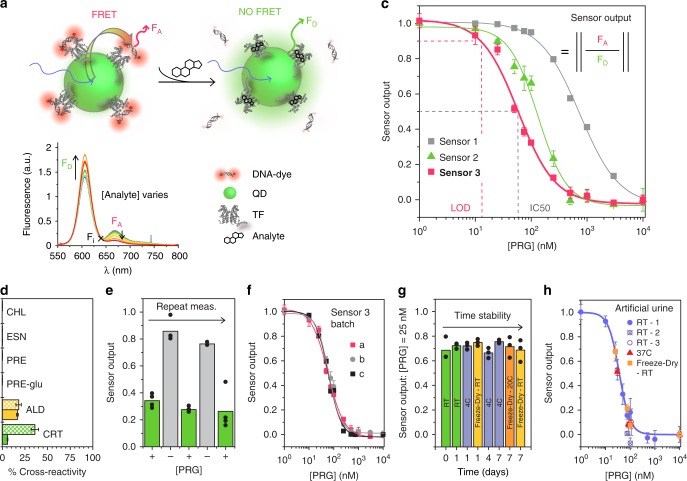
Development and validation of an optical progesterone biosensor. **a** Biosensor scheme. **b** FRET occurs when TFs are bound to DNA resulting in less donor emission and increased acceptor (F_A_). Sensor output is a normalized ratio of acceptor to donor emission. F_i_ = isofluorescence point. **c** Progesterone sensor response. Sensor 1, sensor 2, and sensor 3 described in text. **d** Cross-reactivity to related steroids using sensor 3 with SRFT1 (cross-hatched) and SRTF1_MUT1 (solid). **e** Repeated cycles of progesterone exposure using sensor 3. **f** Reproducibility of different manufactured batches. Batch a and b are derived from 2 different batches of QDs two SRTF1 purifications. **g** Storage stability of sensor 3 in the dark: room temperature (RT), 4 °C, or lyophilized and stored at −20 °C or at RT. **h** Sensor 3 in artificial urine: artificial urine (containing PRG) added at RT or at 37 °C or sensor lyophilized recovered in artificial urine and PRG added in artificial urine. Sensor 3 at RT-1 was tested at 10 different PRG conditions. The fit is of the curve (blue line) is only for this sensor output. Sensors 3 at RT-2, RT-3, 37 °C and freeze-dry were measured at 5 different PRG concentrations. **c**, **f**, **h** Error bars are standard errors over three experiments. Data for reported means in panels **c**, **e**, **f**, **g**, and **h** are available in the Source Data file.

We have developed a framework for coupling the molecular transduction mechanism of aTFs to an optical output (Fig. 3a). Importantly, this approach can be used to develop optical biosensors with any aTF for which a cognate binding site is known. Our framework uses QD-TF-FRET. Quantum dots are widely used in bioimaging and biosensing and provide high photostability, color tunability, and abundant surface area for biofunctionalization^36^. In our approach, QD FRET donors are decorated with purified TFs and a DNA oligonucleotide with the TF binding site is conjugated to a FRET acceptor. TF-hormone binding alters TF-DNA binding resulting in changes in fluorescence. When TFs are bound to the DNA probe, the donor and acceptors are close enough to enable energy transfer. FRET reduces the emission intensity from the QD (donor fluorescence intensity, F_D_) and increases emission intensity from the acceptor (acceptor fluorescence intensity, F_A_, Fig. 3b). When TFs are not bound to the DNA probe FRET is reduced leading to increased F_D_ and decreased F_A_. Sensor output is a normalized ratio of acceptor to donor emission and measuring this ratio increases signal relative to either F_A_ of F_D_ alone. We describe here the comprehensive characterization and validation of this framework through the development of a QD-TF-FRET biosensor.

### An optical progesterone biosensor

We used our QD-TF-FRET framework to develop an optical biosensor for progesterone based on SRTF1. Specifically, CdSe/CdS/ZnS QDs were coated via a biphasic ligand exchange with a zwitterionic polymer containing histamine groups for chelation-based binding to the QD surface^37^. The SRTF1 gene was cloned with a C-terminal 6x His-tag, purified, and self-assembled onto the QD surface at specified average ratios of TFs to QD. Oligonucleotides containing SRTF1 binding sites were labeled with Cy5 as the FRET acceptor and combined with QD-TFs at specified ratios of DNA to QD. The QD emission spectrum overlapped the Cy5 excitation spectrum to enable FRET (Supplementary Fig. 4).

As a first sensor test (sensor 1), oligonucleotides containing the strong SRTF1 binding site upstream of the SRTF1 promoter were used. SRTF1 was combined with QDs at a ratio of 4 TFs per QD and oligonucleotides were combined with QD-TFs at a ratio of 18 oligos per QD. The fluorescence emission of QD-TF/DNA in aqueous solution was immediately measured, using a plate reader, as a function of varying concentrations of progesterone. We observed a clear and reproducible dose-dependent decrease in sensor response with increasing progesterone (Fig. 3c), corresponding to progressive unbinding of SRTF1 from the oligonucleotide and decreased FRET. This sensor response was observed as quickly as it could be measured. As a negative control, we tested the same sensor configuration with an oligonucleotide containing a scrambled binding site and no response was observed (Supplementary Fig. 5). The response of sensor 1 exhibited a broad linear range over more than a log of progesterone concentrations. A fit to a Hill function produced an IC_50_ of 738 nM and a LOD of 53 nM (Supplementary Table 3). Sensor 1 thus demonstrated proof-of-concept for a quantitative progesterone sensor based on a newly characterized bacterial aTF.

While sensor 1 was capable of detecting progesterone at nanomolar levels, we sought to improve this sensitivity. The QD-TF-FRET framework provides several parameters and alternative configurations for sensor optimization (Supplementary Fig. 6). An initial analysis identified three key parameters governing sensitivity. First, as expected, the affinity of the aTF for the analyte is one parameter. Second, consistent with previous reports^38^, our modeling predicted that altering the affinity of the aTF to the oligo would modulate overall sensor sensitivity, with decreasing oligo affinity resulting in increasing overall sensitivity to analyte (Supplementary Fig. 6). This parameter is readily modified in our sensor strategy by altering the aTF binding site. This parameter is not available to antibody-based affinity sensors. Third, we predicted that altering the ratio of TFs conjugated to QDs could improve sensitivity. This is consistent with reports demonstrating that decreasing the density of antibodies bound to particles improves the sensitivity of immunoassays^39^.

We used these predictions to develop progesterone sensors with improved sensitivity. First, we modified the oligonucleotide to contain a binding site with 2 mutated bases in one half of the palindrome to generate sensor 2 (Supplementary Fig. 7). BLI confirmed the lower affinity of SRTF1 to this modified sequence (Supplementary Table 2, Supplementary Fig. 8). As predicted, this resulted in a sensor with substantially improved sensitivity with an IC_50_ of 133 nM and an LOD of 35 nM (Fig. 3, Supplementary Table 3, Supplementary Fig. 5). We then modified sensor 2 to create sensor 3 by decreasing the ratio of TFs to QD to 1:1 while maintaining the 18:1 ratio of oligonucleotides to QD-TF complexes. This further improved sensitivity to an IC_50_ 57 nM and an LOD of 15 nM (Fig. 3, Supplementary Table 3, Supplementary Fig. 5). Sensor 3 also displays an expanded linear range over nearly two logs of progesterone concentration. While we anticipate further improvements to sensitivity with additional tuning of parameters these initial changes result in an order of magnitude increase in sensitivity sufficient to detect progesterone levels in urine and plasma of pre-menopausal women (Supplementary Table 4)^40^.

BLI characterization of SRTF1 indicated that our sensor would exhibit specificity to progesterone. We confirmed this by testing the cross-reactivity of sensor 3 to structurally and physiologically related compounds. As expected, no sensor response was observed when the sensor was exposed to either cholesterol or estrone (Fig. 3d, Supplementary Fig. 9). We also tested our sensor against pregnanediol, which is highly structurally similar to progesterone (Supplementary Fig. 2), and pregnanediol-glucuronide. Again, no cross-reactivity was observed to either metabolite (Fig. 3d, Supplementary Fig. 9). Our sensor is thus a direct sensor of progesterone. Consistent with the BLI results, we observed 36% cross-reactivity to cortisol, and 18% cross-reactivity to aldosterone (Fig. 3d, Supplementary Table 5, Supplementary Fig. 9).

Published reports have demonstrated the ability to dramatically alter the specificity profile of aTFs with a combination of random mutagenesis, directed evolution, and targeted protein modifications^41^. These methods can be used to further increase the specificity of SRTF1-based sensors to progesterone. Thus, we have generated a mutant library of SRTF1. As a first step in characterizing this library, 10 mutants were randomly selected and characterized with a reporter gene assay (Supplementary Fig. 10). One mutant, SRTF1_MUT1, demonstrated a relative decrease in sensitivity to cortisol. Sequencing of the SRTF1_MUT1 gene revealed two mutations located in a region of SRTF1 with the potential to contain the steroid binding domain (Supplementary Fig. 11). We constructed a version of sensor 3 using SRTF1_MUT1 and characterized its response to the panel of steroids. Remarkably, we observed a 6-fold decrease in cross-reactivity to cortisol of 5.7%, with no change in cross-reactivity to other tested sterols (Fig. 3d, Supplementary Table 5, Supplementary Fig. 12). While only preliminary, and based on a randomly selected handful of mutants, these results suggest that directed evolution can further improve both the specificity and sensitivity of SRTF1 to progesterone.

A key advantage of aTF sensors relative to antibodies is the potential for multiple and continuous measurements. This stems from the fact that no secondary assays are required that result in fixation of analyte binding. All interactions associated with the QD-TF-FRET sensor are reversible. As an initial demonstration of this capability, we tested sensors with repeated cycles of progesterone exposure and removal by overnight dialysis (Supplementary Fig. 13). Measurements were performed after each exposure and dialysis. The results confirm that repeated exposures to the same progesterone concentration resulted in nearly identical sensor output (Fig. 3e, Supplementary Fig. 14). While greater variability was observed between measurements after dialysis, this was likely due to limitations in the ability of dialysis to completely remove progesterone. These data confirm the ability of our sensor to reliably measure repeat exposures of progesterone without the need for surface immobilization or additional procedures to reverse binding.

The development of practical devices requires manufacturing and storage reproducibility. To assess this, we tested three independent sensor batches constructed over the span of 6 months. In addition, the sensors were developed from two independent purifications of SRTF1, and a QD with a modified surface treatment and emission spectrum was used for the third sensor batch. As shown in Fig. 3f, the sensor responses to progesterone for all three batches were identical. We further tested the stability of our sensors to storage in different conditions: room temperature, at 4 °C, and at room temperature after freeze-drying for up to a week. In all cases, we observed consistent responses when sensors were retrieved or rehydrated at all tested progesterone concentrations (Fig. 3g, Supplementary Fig. 15). We also tested long-term storage for ten months after freeze-drying. Rehydration restored full sensor responsiveness (Supplementary Fig. 15) although altered dose-response characteristics would necessitate re-calibration. These results are consistent with reports demonstrating the stability of cell-free gene circuits containing transcription factors for over a year^15^.

### Point-of-care urine testing

Progesterone sensing has applications in fertility planning. The detection of surges in luteinizing hormone (LH) and the rise in estrogen prior to the LH surge predict but do not verify ovulation^42^. Detecting progesterone surges confirms ovulation but typically requires blood testing^34,43,44^. Two at-home urine tests are currently being marketed^43,45^ to measure pregnanediol-glucuronide. However, pregnanediol-glucuronide levels are more variable than progesterone levels, and up to 12% of women do not metabolize progesterone sufficiently to produce detectable amounts^34^. To our knowledge, no urine test for the direct detection of progesterone is available. As a first test of our sensor for urine testing, we measured responses to varying concentrations of progesterone in artificial urine. We tested three different conditions using sensor 3: artificial urine at room temperature, artificial urine warmed to body temperature, and sensors freeze-dried and then rehydrated in artificial urine at room temperature. In all cases, we see responses identical to buffer, verifying that common interferents in urine do not affect the sensing of progesterone by the sensor (Fig. 3h).

**Fig. 4:**
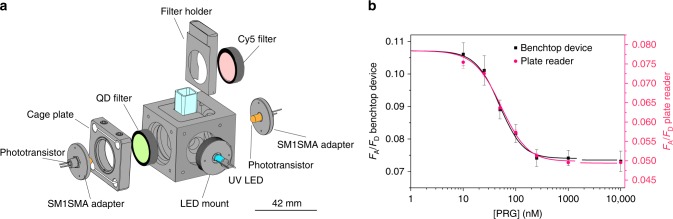
Low-cost portable reader. Proof-of-concept device demonstrating point-of-care use built using inexpensive off-the-shelf electronics. **a** Exploded view of device. The sensor is excited by a UV LED. Filters are used to isolate QD and Cy5 fluorescence emissions, which are then detected by two phototransistors. Scale bar = 42 mm. Full schematic in Supplementary Material. **b** Sensor accuracy using the low-cost prototype device matches accuracy using a high-end plate-reader. Error bars are standard errors over three experiments. Data for reported means in panel B are available in the Source Data file. CAD files for panel **a** cage plate, LED mount, fiber adapter plates, and cuvette holder were purchased from Thorlabs.

Use of our sensor at home or at point of care requires a portable electronic reader. As proof-of-principle, we built a prototype device using inexpensive off-the-shelf detection electronics (Fig. 4a, Supplementary Fig. 16). A 10 mW UV LED controlled by an Arduino was used for excitation. Two phototransistors amplified using a standard low-voltage common emitter circuit detected fluorescence emissions. A 600 nm bandpass filter was used to isolate QD fluorescence emission while a 665 nm long pass filter was used to isolate Cy5 emission (Supplementary Fig. 16). Despite its simplicity, tests of the progesterone sensor in this low-cost device confirmed the same degree of sensor accuracy as with a state-of-the-art laboratory plate reader (Fig. 4b). This proof-of-principle device can be reduced in size and cost through printed circuit board fabrication for the electronics and 3D printing for the housing. Any similar device could be used for low-cost sensing with any bacterial aTF sensor developed with the QD-TF-FRET optical biosensor framework.

## Discussion

Together, our data provide the first demonstration of a microbial screening-to-sensor approach to develop new sensing devices for specific target analytes. The progesterone sensor provides proof-of-concept for a class of sensors based on bacterial aTFs. It demonstrates sensitivity, reproducibility, stability, and signal strength applicable for point-of-care use. It also possesses two key advantages over current antibody-based assays. First, the intrinsic transduction mechanism of aTFs enables real-time and repeat sensing. Our QD-TF-FRET framework converts this transduction to an optical readout. Methods for converting this transduction into a direct electrical readout could also be developed. Second, bacterial proteins are inexpensive to produce, modify, and evolve. The specificity of the progesterone sensor could be further improved through directed evolution^41^, also providing an opportunity to evolve variants specific to other steroids. More broadly, the identification of a progesterone sensing aTF highlights the diverse and largely untapped reservoir of biorecognition elements latent within bacteria. To more fully capture this diversity, our approach can be extended to uncharacterized and unculturable organisms with metagenomic screening^12^, and expanded to target other biorecognition elements, including redox enzymes for electrochemical sensors^1,46,47^. Our results thus provide a paradigm for the targeted development of a diverse range of sensor devices.

## Methods

### Materials

All DNA oligonucleotides were purchased from IDT Technologies. Progesterone (PRG), cholesterol (CHL), cortisol (CRT), aldosterone (ALD), estrone (ESN), 5β-pregnane-3ɑ,20-ɑ-diol (PRE), 5β-pregnane-3ɑ,20-ɑ-diol-glucuronide (PRE-Glu), and lysozyme were bought from Sigma Aldrich. Artificial urine DIN EN1616:199 was bought from Pickering Laboratories. RNAprotect Bacteria Reagent was bought from Qiagen. Proteinase K was bought from Roche.

Cadmium oxide (CdO; 99.95%, Alfa Aesar), sulfur (99.95%, ACROS Organics), 1-octadecene (ODE; 90% ACROS Organics), and oleylamine (80–90%) were bought from Fisher Scientific and used as purchased. Zinc acetate (Zn(Ac)_2_, 99.99%), selenium pellets (Se, 99.99%), trioctylphosphine (TOP, 97%), trioctylphosphine oxide (TOPO, 99%), oleic acid (OA, 90%), poly(isobutylene-*alt*-maleic anhydride)–6000 g mol^−1^ (PIMA), (2-aminoethyl)trimethylammonium chloride, histamine, triethylamine, and HEPES (4-(2-hydroxyethyl)-1-piperazineethanesulfonic acid) were obtained from Sigma-Aldrich. HPLC-grade solvents including hexanes (Fisher Scientific), methanol (Honeywell), anhydrous dimethyl sulfoxide (Sigma Aldrich) and chloroform (J.T. Baker) were bought and used without further purification. HEPES 1× is a solution of 25 mM of HEPES and 150 mM of NaCl, adjusted to pH 7.6.

### Strain selection

*Pimelobacter simplex* strain 6946 was purchased from ATCC and referenced with a corresponding GenBank accession number (CP009896.1). The strain is an obligate aerobe and was grown in media and conditions as recommended by ATCC.

### Strain characterization

To determine the doubling time of the strain, growth curves were generated (Supplementary Fig. 18). All growth curves were done in 100 µL per well volumes in 96 well flat clear bottom black polystyrene TC-treated microplates which were individually wrapped with a lid and sterile (Corning). Measurements were done by an Infinite M200 Pro (TECAN) spectrophotometer at the temperature suited for *P. simplex*. Readings were performed over 96 cycles of 15 min each at 600 nm absorbance with 25 flashes in a 3 × 3 (XY-Line) type reads per well. In between reads there was orbital shaking at 150 rpm frequency for a total of 10 min. To first characterize the growth alone, a ½ serial dilution of 9 concentrations from 0.5–0.0020 OD_600_ nm were prepared in the respective media. Then each concentration was measured as previously described by the TECAN microplate reader and normalized against a media background control in technical triplicate.

### Solvent exposure

Once an appropriate starting cell concentration was chosen, a secondary growth curve was performed to test the toxicity levels of the solvents used to dissolve steroids of interest (Supplementary Fig. 18). Since the steroids used are heavily hydrophobic, they often need to be dissolved in organic solvents such as DMSO or ethanol which are toxic to bacteria at high concentrations. *P. simplex* was incubated under microplate reader conditions previously mentioned at a starting OD determined by the first growth curve with DMSO, ethanol, or H_2_0 at ½ serial dilutions for a total of eight concentrations (50–0.39%) tested in technical triplicate. Two controls were included per solvent; a positive control without solvent and a media control which allowed for appropriate normalization. Solvent exposure growth curves allowed for choosing the maximum amount of solvent concentration that strains would sustain while maintaining relative viability in order to determine a range of steroid concentrations which could be used.

### Steroid exposure

A tertiary growth curve was performed to test the toxicity levels of steroid specific to *P. simplex* (Supplementary Fig. 18). The strain was incubated under microplate reader conditions previously mentioned at a starting OD determined by the first growth curve and the highest solvent concentration corresponding to the steroid of interest with steroid at ½ serial dilutions for a total of seven concentrations tested in singlet. Testosterone, progesterone, 17β-estradiol, hydrocortisone, and aldosterone were all dissolved in ethanol while estrone was dissolved in DMSO. Three controls were included for each steroid; a positive control with the highest tolerable solvent concentration (%), a positive control with media, and a media control which allowed for normalization. Steroid exposure growth curves allowed for choosing the maximum amount of steroid concentration that *P. simplex* would sustain while maintaining relative viability.

### RNA extraction

Cells were grown in 6 mL volumes of media at the OD, solvent, and steroid concentrations found from the growth curves in 14 mL polypropylene round-bottom tubes. The same controls as in the steroid exposure growth curve were used for setting up RNA extraction samples. The cells were incubated at their corresponding temperature with continuous orbital shaking at 150 rpm until mid-log phase and the beginning of stationary phase from the start of inoculation. Afterward, samples were removed and a 1:1 ratio of RNAprotect Bacteria Reagent was added followed by spinning down at 4 °C for 10 min at 4000 × *g*. Supernatant was removed and the pellet re-suspended in 300 µL of RNAprotect and transferred into 2.0 mL Safe-Lock Tubes. The samples were then spun down once again at 4 °C for 10 min at 10,000 × *g*. Once the supernatant has been removed the samples were placed on ice and ready for RNA extraction. RNA extraction was done by Qiacube set to the RNeasy Protect Bacteria Mini Kit protocol of bacterial cell pellet with enzymatic lysis. Tube A was prepared as described except with the addition of 150 mg mL^−1^ lysozyme and 20 mg mL^−1^ proteinase K all diluted in 1× TE buffer. RNA samples were subsequently quantified using Qubit RNA HS Assay Kit (Thermo Fisher Scientific) and analyzed using a RNA 6000 Pico Kit (Agilent) in a 2100 Bioanalyzer (Agilent). RNA samples were either immediately used for RNA-Seq library preparation or stored long-term at −80 °C.

### RNA-Seq library preparation

After RNA samples have been quantified and analyzed they were DNase treated using a TURBO DNase 2 U µL^−1^ (Thermo Fisher Scientific) and cleaned using Agencourt RNAClean XP SPRI beads (Beckman Coulter). RNA-Seq libraries were then produced from these samples using a slightly modified ScriptSeq v2 RNA-Seq Library Preparation Kit (Illumina) ensuring use of unique index primers through ScriptSeq Index PCR Primers (Sets 1–4) 48 rxns/set (Illumina). Libraries were quantified by both a Qubit dsDNA HS Assay Kit (Thermo Fisher Scientific) and by High Sensitivity DNA Kit (Agilent). The resulting molarity was then used to determine at what concentration samples should be pooled to (either 1, 2, or 4 nM). After a desired pooling concentration was chosen, the samples were diluted to that particular molarity and 2 µL of each sample dilution was added into a single tube and submitted to the Boston University Microarray and Sequencing Resource Core Facility. Whole transcriptome RNA sequencing was performed by a NextSeq 500 (Illumina) at high output (400 M reads) with 75 bp paired end sequencing read length. The data was then analyzed in-house through a proprietary Galagan Lab pipeline.

### RNA-Seq data analysis

Adapter sequences were removed from reads and low quality bases trimmed from both ends using Cutadapt^48^. Reads were aligned to the reference genome with Bowtie2^49^. BAM files were sorted and indexed using Samtools^50^. Transcript assembly and expression quantification was performed using Cufflinks^51^. All resulting raw expression counts were normalized as a group using deseq^52^. A custom matlab script was then used to calculate fold changes of normalized counts for each gene between each sterol exposure experiment and its corresponding vehicle control.

### Protein expression and purification

SRTF1 coding sequence codon-optimized for expression in *Escherichia coli* was ordered from Integrated DNA technologies with appropriate up-stream and down-stream fusion sequences for cloning into the pRham C-His Kan vector (Lucigen), resuspended in 1× TE buffer, and 45 ng heat shock transformed into chemically competent E. cloni 10 G (Lucigen) along with 25 ng of linearized pRham C-His Kan Vector (Lucigen). Cells were grown on LB agar + 50 µg mL^−1^ kanamycin overnight at 37 °C. Resulting colonies were grown in 5 mL LB + kanamycin, plasmid purified using Qiagen Miniprep Kit, and CDS insertion verified through Sanger Sequencing using forward primer rRham F and reverse primer pEtite R (Lucigen).

Protein was expressed in E. cloni 10 G cells grown overnight from a singly colony. Five milliliters overnight LB + kanamycin culture was diluted in 250 mL of fresh LB + kanamycin, then allowed to grow to an OD600 of approximately 0.6 shaking at 37 °C. Two and a half milliliters of filter sterilized 20% w/v rhamnose was then added to induce protein expression, and culture was shaken for 4 h at 37 °C. Culture was removed then removed from the incubator and stored at 4 °C overnight. Culture was then pelleted by centrifugation and frozen overnight.

Protein was purified using the Ni-NTA Fast Start kit (Qiagen). Cells were lysed using 10 mL of the provided Native Lysis Buffer supplemented with 10 mg lysozyme and 250 U benzonase as specified in the manufacturer’s protocol. Pellet was resuspended by stirring and pipetting up and down with a 25 mL graduated pipette. Cells were lysed on ice for 1 h, with gentle swirling every 20 min. Some preparations were highly viscous after the 1 h incubation, likely due to incomplete nuclease activity. Viscous lysates had an additional 250 U benzonase added to them and were incubated for twenty more minutes. All samples treated with additional benzonase had their viscosity reduced to normal after this additional incubation. Lysate was centrifuged at 14,000 × *g* for 30 min at 4 °C. Lysate supernatant was applied to a pre-drained Ni-NTA column and allowed to run through at room temperature. Column was washed three times with 4 mL of 3 mL of Native Wash Buffer (NEB), then eluted twice with 1 mL each of Elution Buffer (NEB).

Protein was present in both elution fractions off the column, so elutions 1 and 2 were pooled for desalting. Protein was either dialyzed against the destination buffer using 0.5–3 mL 3500 MWCO Slide-a-Lyzer (ThermoFisher), twice for 2 h at room temperature with magnetic stirring, then overnight at 4 °C without stirring, or using Amicon Ultra-0.5 centrifugal filters with a 10 kDa MW cutoff. Elutions were added to the column 0.5 mL at a time, spun at 14,000 × *g* at 4 °C until only approximately 75 µL remained, around 30 min. Destination buffer was added to the 0.5 mL mark, and centrifugation repeated. This process was repeated 3 additional times, for a total of 4 times.

Destination buffer for protein used in BLI, iv-ChIP-seq, and QD-TF-FRET assays was Tris-buffered saline pH 7.4. Protein concentration was quantified using a Qubit Protein Assay kit (ThermoFisher), then aliquoted and frozen at −80 °C until used.

### In vitro ChIP-Seq

We developed an in vitro ChIP-Seq assay similar to previously published in-vitro DNA precipitation protocosl^53,54^. *P. simplex* picked from a single colony on M3 Agar was grown in M3 Broth at room temperature with gentle shaking for 3 days until culture was turbid. One milliliter aliquots of culture were pelleted and genomic DNA was extracted using the GenElute Bacterial Genomic DNA kit (Sigma-Aldrich) using the included Gram-Positive Lysis solution and associated protocol adjustments for Gram positive bacteria. Purified gDNA was sheared into approximately 200 bp fragments in 130 μL aliquots using a Covaris S2 sonicator using a duty cycle of 20%, an intensity of 5, 200 cycles per burst, a time of 60 s, and 12 total cycles.

Purification of fragments of an appropriate size was done using AgenCourt AmpPure XP beads. 1.8× volume of bead slurry was added to the sonicated DNA and mixed by pipetting, followed by a five-minute incubation at room temperature. The DNA/slurry mixture was then placed on a magnetic rack for 10 min until all beads were pulled to the side of the tube. Supernatant was aspirated, and beads were washed twice with 200 μL 70% ethanol while still on the magnetic rack. Beads were air dried for 10 min, then resuspended in 100 μL of water, mixed by pipetting, and incubated at room temperature for 10 min. Beads were pulled down by placing on a magnetic rack for 10 min, then the supernatant and eluted DNA was pipetted off and saved. Fragment length was confirmed through 1% agarose gel electrophoresis in 1× TBE.

Fifty nanogram of sheared DNA was mixed with 500 ng purified SRTF1-CHis to a final volume of 500 μL in 1× IPP150 (10 mM Tris-HCl pH 8.0, 150 mM NaCl, 0.1% NP40). Mock ChIP reactions were done by omitting the purified protein. Mixture was incubated at room temperature for 1 h, then 3 μL of 6×-His Tag Monoclonal Antibody His.H8 (Thermofisher Scientific) was added. Mixture was then incubated at 4 °C overnight with rocking.

Incubated mixture was then mixed with 5 μL of Protein G agarose beads (Pierce) that had been washed with 1 mL of IPP150 and pelleted by centrifugation for 2 min at 2000 × *g*. Bead mixture was incubated on a rocking platform for half an hour at 4 °C, then an hour and a half at room temperature. Beads were washed 3 times with 1 mL of IPP150, mixed by rocking each time. Wash buffer was removed by pelleting beads at 2000 × *g* for 2 min and pipetting off supernatant. DNA was eluted by adding 150 μL of “Elution From Beads” buffer (50 mM Tris-HCl pH 8.0, 10 mM EDTA, 1% SDS) to pelleted beads and incubating at 65 °C for 15 min. Elution was repeated with a 5 min 65 °C incubation. Elutions were pooled, then purified using a QIAquick PCR Purification Kit (Qiagen), eluting with 30 μL of buffer EB.

Barcode adapters were ligated to immunoprecipitated DNA using the NEBNext Ultra II DNA Library Prep Kit for Illumina. As starting gDNA had been pre-sheared before immunoprecipitation, size selection was performed after 10 cycles of PCR barcoding. Library size and integrity was validated with an Agilent DNA 1000 Kit on an Agilent Bioanalyzer.

Library was sequenced on an Illumina NextSeq 500 to a depth of 4–10 M reads per sample. Illumina adapter sequences were removed with Cutadapt, and reads were aligned to the *P. simplex* VKM Ac-2033D genome CP009896.1 using Bowtie2.

### BioLayer interferometry

Single stranded DNA oligos containing SRTF1 binding sites or control sequences were ordered from IDT. Forward strands with 5′ biotin were annealed to reverse strands by mixing to a final concentration of 10 µM in Annealing Buffer (10 mM Tris-HCl pH 8.0, 50 mM NaCl), heating to 95 °C, then slowly letting them cool to room temperature.

All BLI steps were performed in 1× BLI Buffer (28 mM Tris-HCl pH 8.0, 5 mM MgCl, 4.25% Glycerol, 25 mM NaCl, 1.67 mg mL^−1^ BSA) at 30 °C in a ForteBio OctetRed96. For DNA association assays, SA tips were baselined in buffer, then dsDNA was loaded on by dipping into wells containing 75 nM DNA in buffer. DNA-coated tips were baselined again in buffer, then dipped in buffer containing varying concentrations of SRTF1 and allowed to reach equilibrium. Tips were then dipped in buffer for complex dissociation. Dissociation of DNA from the tip was controlled by subtracting the trace from a DNA-coated tip that was not exposed to SRTF1. Trace Y-Axes were aligned to the last 5 s of the second baseline step, and noise removed by Savitzsky-Golay filtering. Association and dissociation data were fit to a Mass Transport model.

Hormone-induced dissociation was assayed using BLI by dipping DNA-coated SA tips in buffer containing 150 nM SRTF1 and allowing the reaction to reach equilibrium. Complex was dipped in buffer containing varying amounts of hormone. Curve traces for each sensor were rescaled such that the binding level at equilibrium was equal to 1.

### Quantum dots synthesis

Core/shell/shell quantum dots were made using our previously reported procedure^55^ and is described briefly here. The precursors used for this synthesis included: 0.2 M Cd(OA)_2_, 0.2 M Zn(OA)_2_, 0.2 M sulfur in ODE, and 1 M TOP:Se. For the sulfur and selenium precursors, the appropriate amount of anion was weighed and dissolved into either ODE or TOP at the desired concentration with gentle heating. Once the solutions were fully dissolved, the precursors were heated under vacuum at 120 °C for at least 1 h before use to remove traces of water. For Cd(OA)_2_ and Zn(OA)_2_, CdO or Zn(Ac)_2_ were weighed and added to oleic acid at a 1:4 molar ratio. The solutions were heated under vacuum at 120 °C until fully dissolved and diluted to a final concentration of 0.2 M with ODE. All precursors were stored under argon at room temperature. Both Cd(OA)_2_ and Zn(OA)_2_ are waxy solids at room temperature and were therefore heated to 120 °C for use in the QD synthesis.

For nucleation of CdSe cores, we used an air-free hot injection method. In brief, 1 g of TOPO, 8 mL of ODE, and 1.9 mL of 0.2 M Cd(OA)_2_ were loaded into a 100 mL round bottom flask (rbf) and placed under vacuum at room temperature for 30 min. The flask was heated to 80 °C and degassed by backfilling with argon and switching back to vacuum 3× over the course of 1 h. Once the solution had been sufficiently degassed, the flask was placed under active argon flow and heated to 300 °C. In an argon-filled glovebox, we pre-mixed 4 mL of 1 M TOP:Se, 3 mL of oleylamine, and 1 mL of ODE for injection into the Cd solution at 300 °C. The reaction temperature was set to 270 °C. After 3 mins, the flask was taken off of the heating element and allowed to cool to room temperature. The CdSe cores were precipitated from solution under air-free conditions using ethanol and methanol and re-dispersed in hexanes.

For shelling, a successive ion layer adsorption reaction (SILAR) was used as previously described. In brief, 5 mL ODE and 5 mL oleylamine were added to a 100 mL rbf and heated under vacuum at 120 °C for 1 hr before 200 nmol of CdSe cores in hexanes were added to the flask, and the hexanes removed via low-pressure evaporation. For each shell material, a single monolayer, defined by the lattice constant of each material, was added at a time. The amount of precursor needed to add each monolayer was calculated on a volume basis using the density and lattice constants for wurtzite CdS and ZnS. For the CdS shell, 1 monolayer of CdS was added. The first Cd addition was added dropwise at 160 °C to the core solution under argon and annealed for 2.5 h. The temperature was then increased to 240 °C and the corresponding amount of sulfur precursor added dropwise and annealed for 1 h. All additional monolayers were added and annealed at 240 °C. After CdS shelling, 2 monolayers of ZnS were added in a similar fashion. After 2 full monolayers of ZnS were added, an additional layer of Zn was added to ensure that the QD surface was Zn-rich.

### Polymer synthesis

The polymer capping the QDs (P1) was synthesized using a slightly modified version of a previously reported procedure^56^. In a typical experiment, 180 mg of PIMA (poly(isobutylene-*alt*-maleic anhydride), 6000 g mol^−1^, 0.03 mmol, 1 equivalent) was dissolved in 3 mL anhydrous dimethyl sulfoxide at 45 °C. In parallel, 116 mg (2-aminoethyl)trimethylammonium chloride (0.66 mmol, 22 equivalents), 73 mg histamine (0.66 mmol, 22 equivalents), and 193 µL triethylamine (1.39 mmol, 46 equivalents) were dissolved in 1.5 mL anhydrous dimethyl sulfoxide at 50 °C. After complete dissolution of both solutions, the solution containing the amines was added with a syringe to the PIMA solution. The reaction was kept overnight at 45 °C. The polymer was purified by several precipitations in ethyl acetate. A white powder was obtained with 67% yield. 1 H NMR (500 MHz, D_2_O): δ(ppm) = 8.33 (s, 0.35 H, imidazole), 7.14 (s, 0.52 H, imidazole), 3.61 (0.63 H), 3.37 (2.24 H), 3.08 (s, 5.14 H, quaternary amine), 2.82 (1.13 H), 2.49 (1.26 H), 2.15 (1.01 H), 2–1 (m, 2.45 H), 0.86 (m, 6 H, 2CH_3_). FTIR shows the disappearance of the C=O stretch band of the anhydride at 1770 cm^−1^ and appearance of the C=O stretch of carboxylic acid and amide bond at 1710 cm^−1^ and1650 cm^−1^, respectively.

### Ligand exchange

QDs were transferred into water by capping their surface with the polymer P1. In a typical experiment, 475 µL QDs ([QD] = 3.0 µM, 1.4 nmol) were dissolved in 600 µL of chloroform. In parallel, 560 µL of P1 at 10 mg mL^−1^ in DMSO was diluted in 560 µL chloroform. The solution of P1 was added to the QD dispersion and the reaction proceeded overnight with vigorous stirring. The next day, 0.5 mL of 0.1 M NaOH was added and the dispersion quickly shaken by hand. The QDs nicely transferred to the upper water phase. The water phase was extracted and centrifuged at 3800 × *g* for 1 min. Then the supernatant was filtered through 100 nm PVDF and washed 3 times with 0.1 M NaHCO_3_ with a 100k ultra-centrifugal filter. QDs were recovered in 0.1 M NaHCO_3_ at a concentration around 5 µM.

### Sensor assembly

For a typical experiment, using a molar ratio of QD/TF/DNA = 1/4/18: 275 µL QDs at 0.15 µM in 1× HEPES with 1% BSA, were mixed with 275 µL SRTF1-his6 at 0.6 µM in 1× HEPES, at room temperature for 45 min. Double-stranded DNA labeled with a Cy5 fluorescent probe at the 3′ and 5′ ends (275 µL, 2.7 µM in 1× HEPES,) was added to the mixture. After 30 min, 220 µL of 1× HEPES, and 330 µL of 5× binding buffer (25 mM MgCl_2_, 25% glycerol, and 250 mg L^−1^ Invitrogen™ UltraPure™ Salmon Sperm DNA in 0.1 M Tris-HCl) were added and the mixture incubated for 15 min at RT.

### Plate reader measurements

Fluorescence measurements were recorded on a Horiba Nanolog spectrofluorometer equipped with a plate reader. Absorption spectra were recorded using a Nanodrop 2000c. Fifty microliter of the sensor (QD/TF/DNA) was split in 3 × 9 centrifuge tubes to which 10 µL of progesterone at the desired concentration is added. As such, the final concentration of QD/TF/DNA for the measurements is 25 nM/100 nM/450 nM. A 384-well plate was filled with 60 µL of each solution. The fluorescence intensity was monitored on a spectrofluorimeter from 535 nm to 800 nm with excitation at 400 nm and a 450 nm long-pass filter before the emission detector. Fluorescence intensity was spectrally deconvolved into FRET donor and acceptor components and total intensity used to calculate normalized F_A_/F_D_. Ratiometric analysis using single wavelength point measurements of F_A_ and F_D_ displayed equivalent accuracy to full deconvolution.

### Stability assays

For the stability assays, QDs, TF, and DNA were mixed together and stored in the conditions described (e.g., RT, at 4 °C, or in the freezer following lyophilization); in each case the sensors were protected from light to prevent photobleaching. HEPES 1× and 5× binding buffers were added to the sensor components before starting the progesterone titration. Lyophilized sensors were first recovered in ultra-pure water (same volume as sublimated during lyophilization process), then HEPES 1× and 5× binding buffer were added before the progesterone titration.

### Cross reactivity calculation

Cross-reactivity is calculated with the following equation^57^:

$${\mathrm{\% Cross}}\,{\mathrm{reactivity}}\,=\,\frac{{{\mathrm{IC}}_{50}{\mathrm{of}}\,{\mathrm{analyte}}}}{{{\mathrm{IC}}_{50}{\mathrm{of}}\,{\mathrm{cross}}\,-\,{\mathrm{reactant}}}}$$

Cross-reactivity assays were performed using the sensor 3 configuration in HEPES 1× with SRTF1 and the mutant SRTF1_MUT1.

### Limit of detection calculation

The detection limit is the smallest concentration or absolute amount of analyte that has a signal significantly larger than the signal arising from a reagent blank. Mathematically, the limit of detection in the signal domain (*L*_D_) is given by:

$$L_{\mathrm{D}}\,=\,{\mathrm{mean}}_{{\mathrm{blank}}} - 3.3 \times {\upsigma}_{{\mathrm{test}}}$$

where *mean*_*blank*_ is the mean signal for a reagent blank and *σ*_*test*_ is the pool standard deviation for all test samples in the dilution series, calculated as^58^:

$${\upsigma}_{{\mathrm{test}}} = \sqrt {\frac{{\mathop {\sum}\nolimits_{i = 1}^m {\sigma _i^2} }}{m}} $$

where *σ*_*i*_ is the standard deviation in signal intensities for *n* replicates of the *i*th test concentration, with a total of *m* different test concentrations.

The limit of detection (LOD) was calculated using the parameters of the fit with the non-linear equation for *y* = *L*_D_:

$${\mathrm{LOD}}\,=\,{\mathrm{IC}}_{50}\,\times\,\root {p} \of {{\frac{{A_1 - A_2}}{{L_{\mathrm{D}} - A_2}} - 1}}$$

The 95% Confidence Interval was calculated using Origin Pro Software.

### Artificial urine assays

Artificial urine composition: pH 6.6 ± 0.1, urea 25.0 g L^−1^, sodium chloride 9.0 g L^−1^, disodium hydrogen orthophosphate anhydrous 2.5 g L^−1^, potassium dihydrogen orthophosphate 2.5 g L^−1^, ammonium chloride 3.0 g L^−1^, creatinine 2.0 g L^−1^, sodium sulfite hydrated 3.0 g L^−1^.

For the artificial urine assays, QDs, TF, and DNA were mixed together in 1× HEPES before artificial urine and artificial urine + PRG was added such that 50% of the final volume comprised artificial urine.

Artificial urine at 37 °C: the sensor was assembled at RT then artificial urine and artificial urine + PRG were added at 37 °C.

For tests in artificial urine following lyophilization, QDs, TF and DNA were assembled in 1% BSA for QDs and ultra-pure water (no salts) and lyophilized. The sensor was recovered in artificial urine (same volume as sublimated during lyophilization process). Then artificial urine + PRG was added to the sensor.

### Portable electronic reader

A UV-LED (LED405E, 10 mW, Thorlabs) was attached to an SM1-threaded LED mount (S1LEDM, Thorlabs) that was screwed into one of the ports of the cuvette holder. The LED was powered with 3.3 V using an Arduino Uno. Two phototransistors (Digi-Key, 751-1057-ND) were used to detect the emitted light, one for each channel. In order to detect the Cy5 emission (670 nm), a 665 nm LP filter (Chromatech, ET655lp) was placed in front of one of the phototransistors to filter the light emitted by the Cy5 dye. In order to detect the QD emission (605 nm), a 600 nm BP filter (Edmund Optics, P/N 84785, 600 nm, FWHM 50 nm) was placed in front of the second phototransistor to filter the light emitted by the QDs.

Each one of the phototransistors was placed in a common-emitter phototransistor circuit (Supplementary Fig. 16). A DC power supply was used to power the circuit (Bk Precision, 1760A). During an experiment, the voltage drop across the phototransistor was monitored with a multimeter (Agilent34410A) that was connected to LabView, which enabled real-time recording of measurements. The device was enclosed in a metal Faraday cage.

The LED was turned on and allowed to warm up for at least 20 min prior to the experiment. One hundred microliter of sensor with a given concentration of progesterone was pipetted into the cuvette. The cuvette was inserted into the cuvette holder such that the 10 mm path length was parallel to the filters. Upon inserting the cuvette, a timer was started. A black cover was placed over the cuvette and a lid placed on the Faraday cage. At *t* = 10, 30 and 50 s, a Labview program was started to begin data collection for a duration of 10 s each. Each 10 s long period of data collection is counted as one technical replicate.

The following equations were used to calculate F_A_/F_D_ for the benchtop device:

$$\frac{{\mathrm{F}}_{{\mathrm{A,}} {c,i}}}{{\mathrm{F}}_{{\mathrm{D,}}{c,i}}}\,=\,R_{c,i}\,=\,\frac{{{\mathrm{Cy}}5_{{\mathrm{signal}},c,i}}}{{{\mathrm{QD}}_{{\mathrm{signal}},c,i}}}$$

where

$$\begin{array}{l}{\mathrm{QD}}_{{\mathrm{signal}},c,i} = \left( {5 - V_{out,cy5,i}} \right)\\ {\mathrm{Cy}}5_{{\mathrm{signal}},c,i} = \left( {5 - V_{out,QD,i}} \right)\end{array}$$

*c* = the concentration of progesterone in the sample *i* = the replicate number for a given concentration (i.e., 1 is the first technical replicate, etc.)

The standard deviation of each ratio is calculated using the following equation:

$$\sigma _{R_{c,i}}\,=\,R_{c,i}\sqrt {\left( {\frac{\sigma _{{\mathrm{QD}}_{{\mathrm{signal,}}{c}}}}{{{\mathrm{QD}}_{{\mathrm{signal}},{{c}},i}}}} \right)^2 + \left( {\frac{{\sigma _{{\mathrm{Cy}}5_{{\mathrm{signal}},c}}}}{{{\mathrm{Cy}}5_{{\mathrm{signal}},{{c}},i}}}} \right)^2}$$

In order to calculate the error bars associated with the average of the Ratio, the following equation was used:

$$\sigma _{Rc} = \frac{{\sqrt 3 }}{3}\sqrt {\left( {\sigma _{Rc,1}} \right)^2 + \left( {\sigma _{Rc,2}} \right)^2 + \left( {\sigma _{Rc,3}} \right)^2} $$

### Reporting summary

Further information on research design is available in the Nature Research Reporting Summary linked to this article.

## Supplementary information

Supplementary Information

Reporting Summary

## Data Availability

All RNA-Seq data that support this paper have been submitted to the Gene Expression Omnibus with the accession number GSE141603. All iv-ChIP-seq data that support this paper have been submitted to the Gene Expression Omnibus with the accession number GSE131041. Source data from which Figs. 2c, e, 3c, e, f, g, h, 4b, and Supplementary Figs. 5, 9, 10, 12, 14, and 15 are generated are located in the Source Data file. Other relevant data are available on request.

## Source Data

Source Data

## Code availability

Custom software, and analysis scripts will be made available on request.
