# Supplementary material for: A progesterone biosensor derived from microbial screening
Source: Nat Commun. 2020 Mar 9;11:1276. doi: 10.1038/s41467-020-14942-5 (PMC7062782; doi:10.1038/s41467-020-14942-5)
Supplement: Supplementary file 1 — Supplementary Information [file 41467_2020_14942_MOESM1_ESM.pdf]

Supplementary Information

for

A Novel Progesterone Biosensor Derived from Microbial Screening

Chloé Grazon[1,2,#], R C. Baer[3,4,#] ,Uros Kuzmanovic[5], Thuy Nguyen[5], Mingfu Chen[5], Marjon Zamani[5], Margaret Chern[6], Patricia Aquino[5], Xiaoman Zhang[5], Sébastien Lecommandoux[2], Andy Fan[5], Mario Cabodi[5], Catherine Klapperich[5,6], Mark W. Grinstaff[1,5,6], Allison M. Dennis[5,6], James Galagan[3,4,5,§]

[#]Contributed Equally
[§]Corresponding Author: jgalag@bu.edu

[1]Department of Chemistry, Boston University, Boston, MA 02215, USA
[2]University Bordeaux, CNRS, Bordeaux INP, LCPO, UMR 5629, F-33600, Pessac, France
[3]Department of Microbiology, Boston University, Boston, MA 02118, USA
[4]National Emerging Infectious Diseases Laboratories, Boston University, Boston, MA 02118, USA
[5]Department of Biomedical Engineering, Boston University, Boston, MA 02215, USA
[6]Division of Materials Science and Engineering, Boston University, Boston, MA 02215, USA

Table of Contents

Supplementary Figures **2**
Supplementary Tables **21**
Supplementary Information References **29**

Supplementary Figures

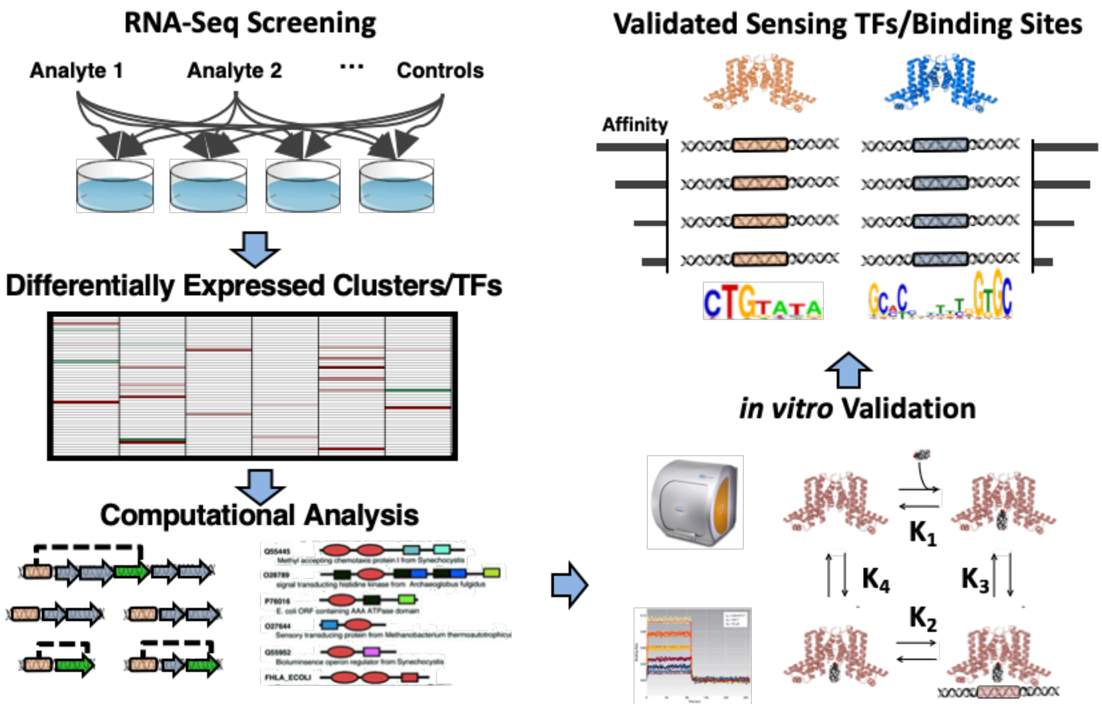

Supplementary Figure 1: Illustration of a combination of genomic and functional screens for the targeted identification, validation, and characterization of TF biosensors for specific analytes.

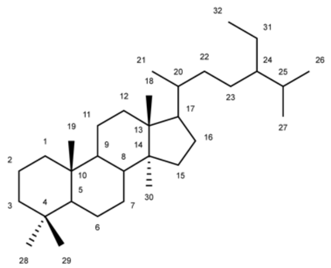

Steroid skeleton
(Carbons 18 and above can be absent)

(PRG)
Progesterone

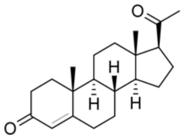

$C_{21}H_{30}O_2$
M: 314.46 g/mol

(ALD)
Aldosterone

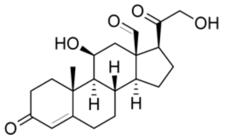

$C_{21}H_{28}O_5$
M: 360.44 g/mol

(PRE-Glu)
5β-Pregnane-3α,20α-diol glucuronide

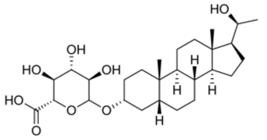

$C_{27}H_{44}O_8$
M: 496.63 g/mol

(PRE)
5β-Pregnane-3α,20α-diol

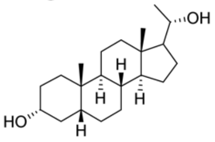

$C_{21}H_{36}O_2$
M: 320.51 g/mol

(CHL)
Cholesterol

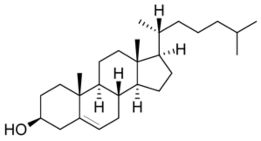

$C_{27}H_{46}O$
M: 386.65 g/mol

(CRT)
Hydrocortisone

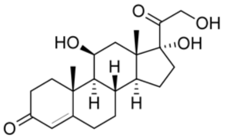

$C_{21}H_{30}O_5$
M: 362.46 g/mol

(ESD)
17β-Estradiol

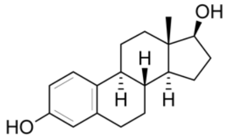

$C_{18}H_{24}O_2$
M: 272.38 g/mol

(ESN)
Estrone

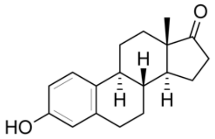

$C_{18}H_{22}O_2$
M: 270.37 g/mol

Supplementary Figure 2: Structures and abbreviations of sterols used in this study.

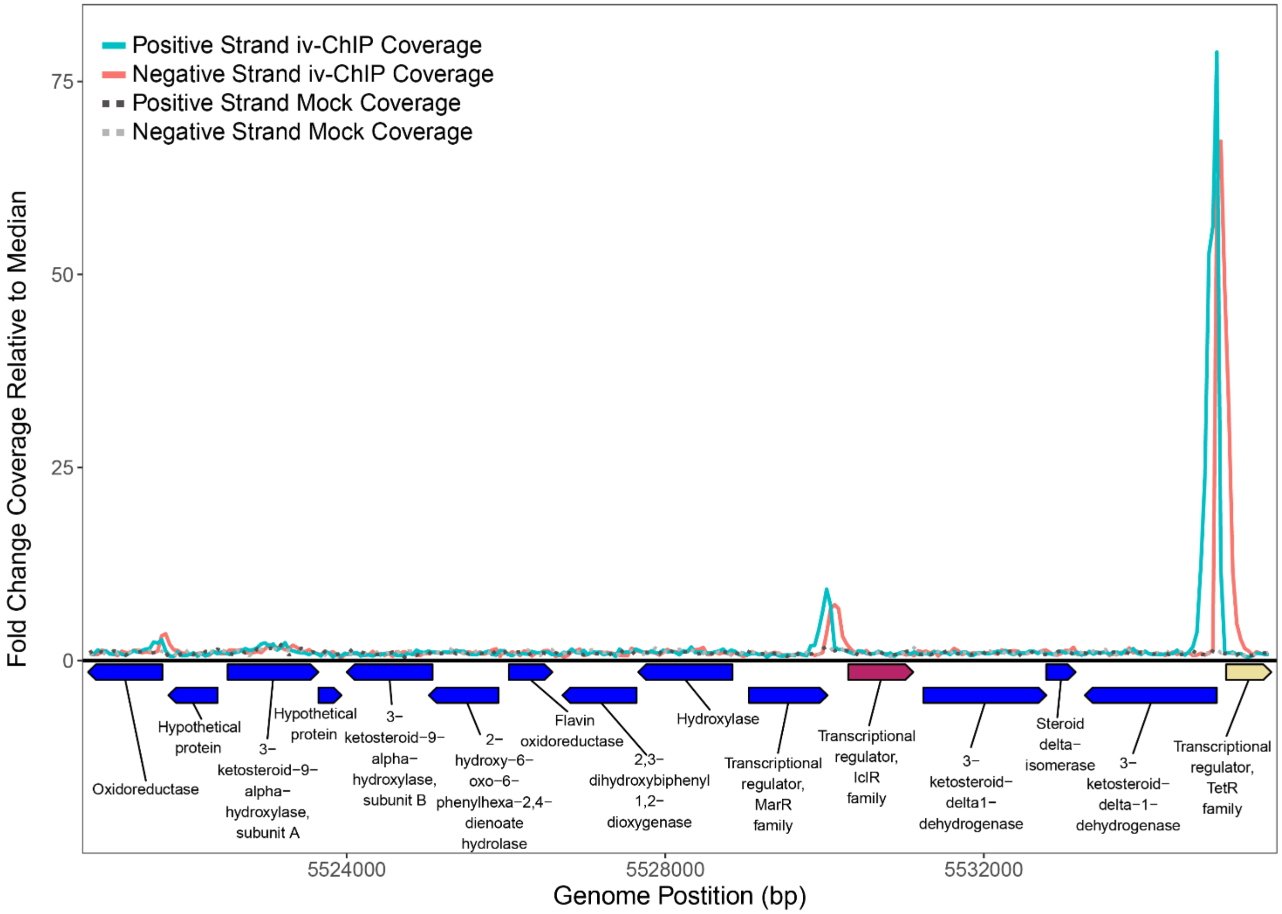

Supplementary Figure 3: *In vitro* ChIP-Seq of SRTF1 (yellow) reveals binding of SRTF1 to binding sites upstream of SRTF1, SRTF2 (maroon), and a predicted oxidoreductase. Binned ChIP reads of an iv-ChIP experiment and a mock iv-ChIP experiment without SRTF1 were normalized against the median coverage across the SRGI and plotted against genome position. ChIP-seq data are available on the Gene Expression Omnibus with accession number GSE131041.

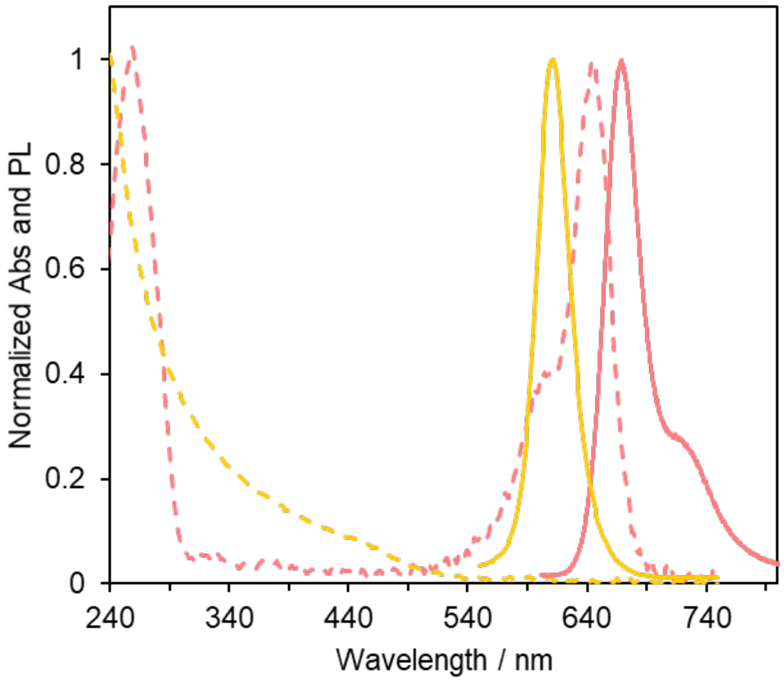

Supplementary Figure 4: Measured absorption (dashed) and fluorescent emission (solid) of QDs (yellow, λ_{ex} = 400 nm) and DNA-Cy5 (red, λ_{ex} = 540 nm) in 1x HEPES, pH 7.6.

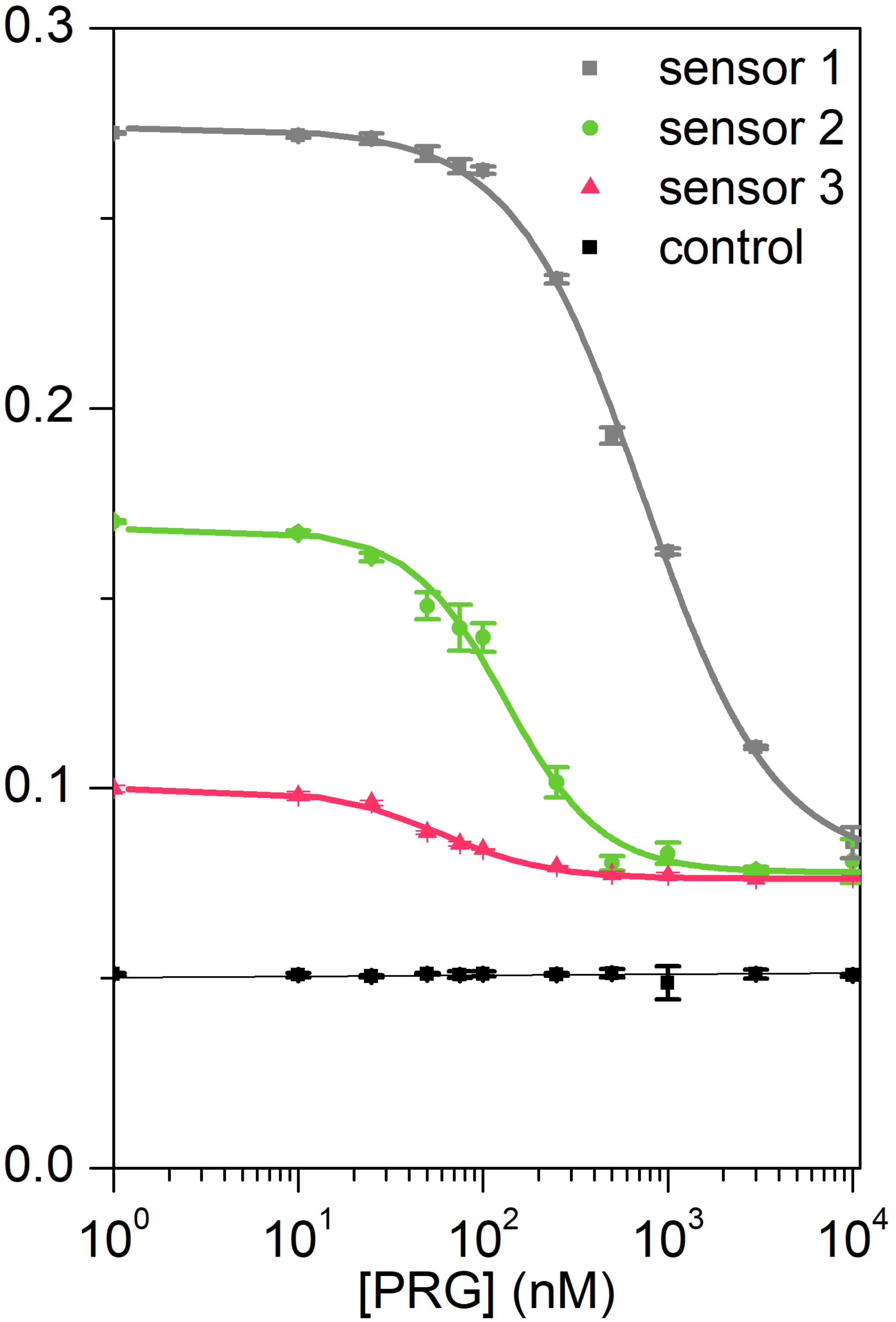

Supplementary Figure 5: Unnormalized dose-response curve for 3 sensors recorded after addition of progesterone from 0 to 10000 nM. Sensor 1, sensor 2, and sensor 3 described in text. Control is performed using the scrambled DNA with QD/TF/DNA ratios = 1/4/18. Error bars are standard errors over three experiments. Data underlying means are available in the Source Data file.

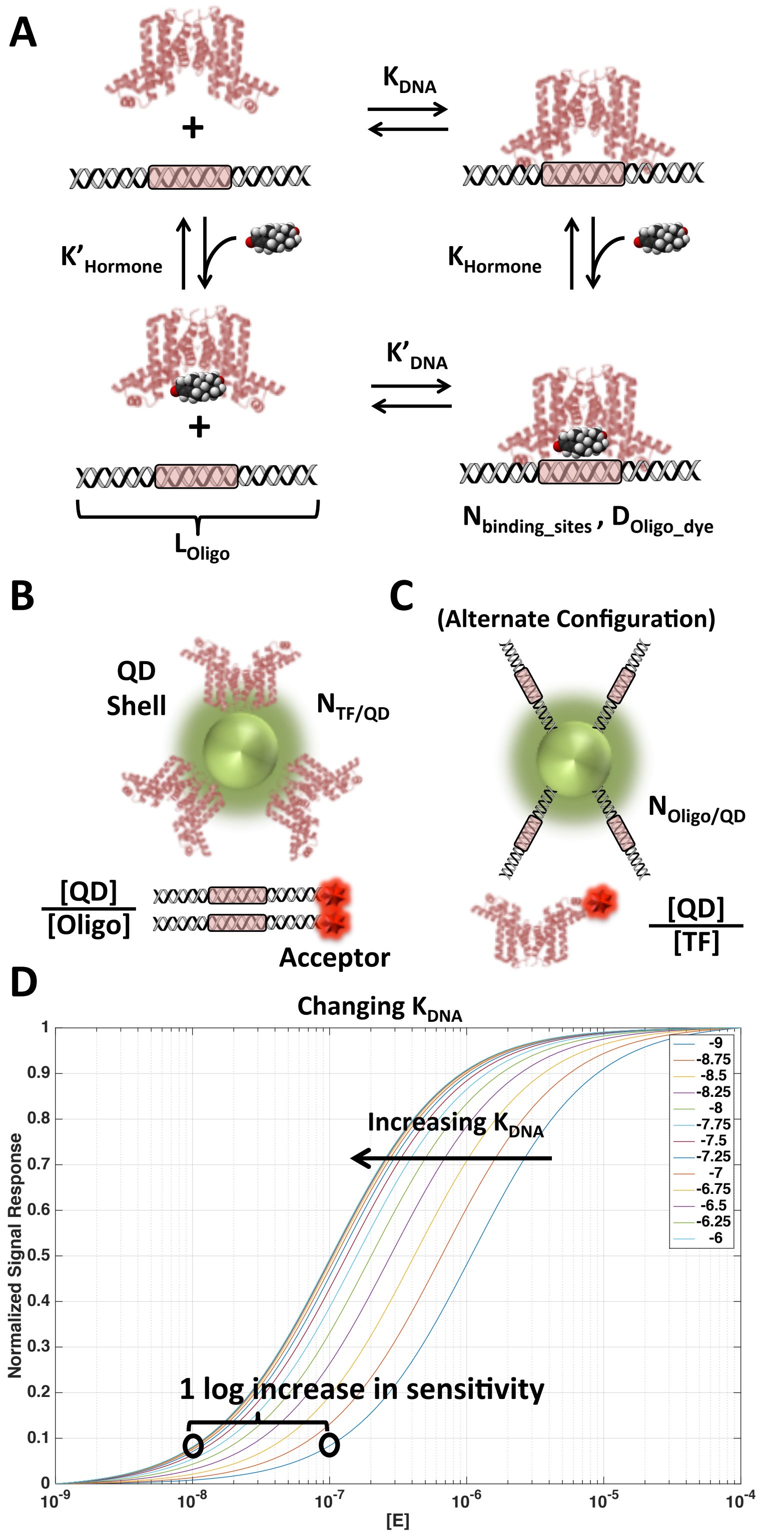

Supplementary Figure 6: Transduction system parameters and configurations. (A) Binding of TF to oligo can be described by a Michaelis-Menton model. (B) Our tested QD-TF-FRET configuration, and (C) an alternative configuration. (D) An initial model of our system predicts that increasing K_{DNA} (i.e., reducing the affinity of the DNA-aTF binding) increases sensitivity.

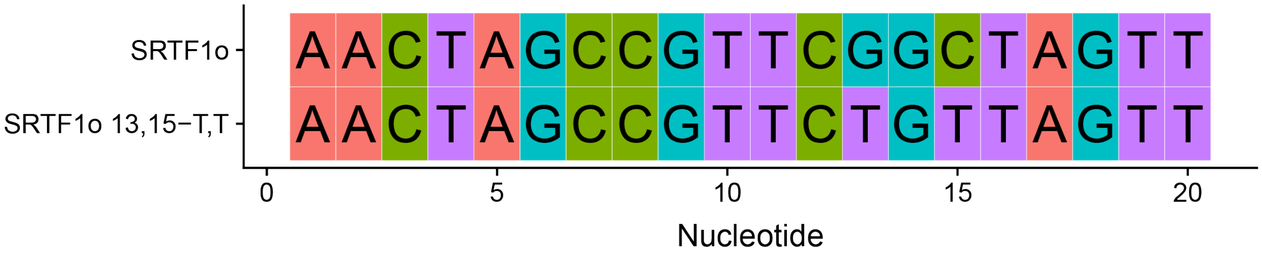

Supplementary Figure 7: Strong binding site for SRTF1 (top, *SRTF1o*) and mutated weaker site (bottom, *SRTF1o* -13,15-T,T) with disrupted palindrome.

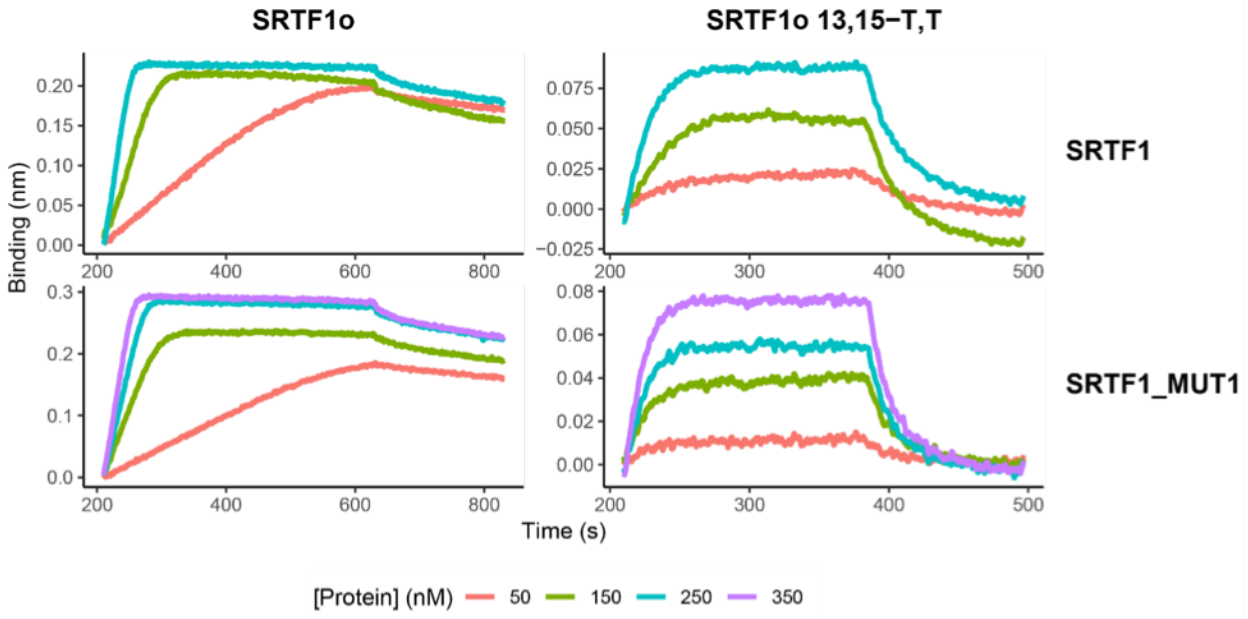

Supplementary Figure 8: BLI binding curves of SRTF1 (50, 150, and 250 nM) and SRTF1_MUT3 (50, 150, 250, and 350 nM) to DNA containing the palindromic binding site upstream of SRTF1 (*SRTF1o*) and the mutated version of this site (*SRTF1o* -13,15-T,T). BLI tips were dipped in 75 nM biotinylated dsDNA, baselined, dipped in buffer containing protein, then dipped in buffer for dissociation once saturation was reached.

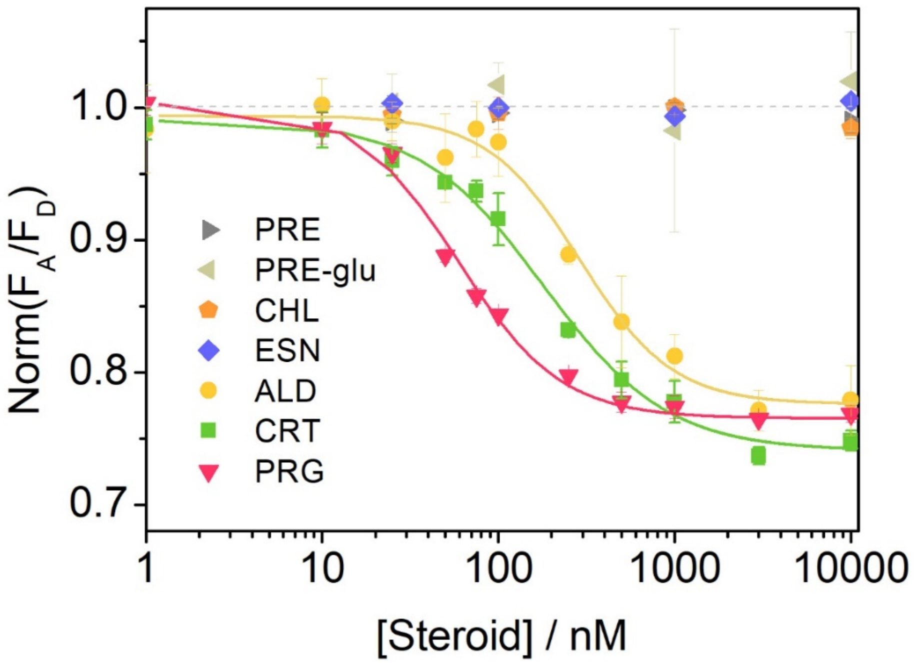

Supplementary Figure 9: Normalized fluorescent dose-response curve of the sensor 3, using SRTF1, in response to 7 different steroids. Error bars are standard errors over three experiments. Data underlying means are available in the Source Data file.

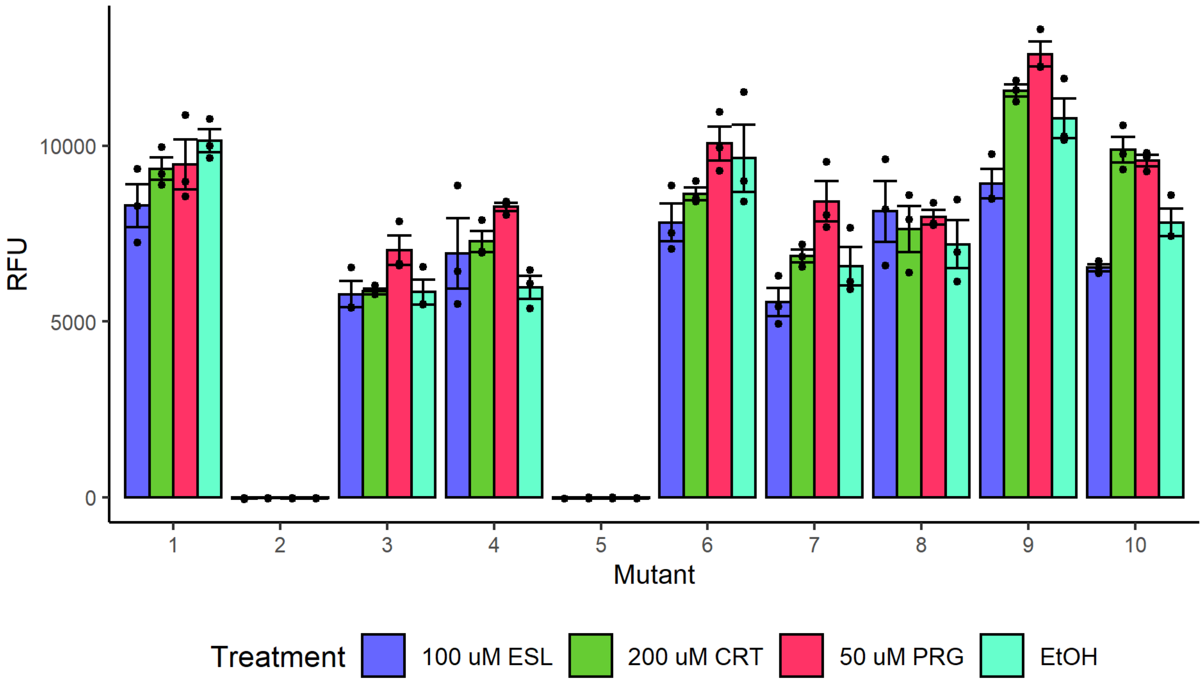

Supplementary Figure 10: SRTF mutant GFP reporter assay with progesterone, cortisol, and estradiol. The strong palindromic binding site for SRTF1 (*SRTF1o*) was placed directly downstream of the J23102 constitutive promoter, which was cloned upstream of GFP and constitutively expressed STRF1 variants. Variants were exposed to concentrations of progesterone and cortisol that elicit equal fluorescent response in the wild-type protein, grown for two hours, then fluorescence was assayed. Variant 3 showed a greater fluorescence for progesterone relative to other sterols or vehicle, and was named SRTF1_MUT1. Error bars are standard errors over three experiments. Underlying data is available in the Source Data file.

```
SRTF1 -       MSSTAERIRPGRSGILAAATRLFATHGVSGTSLQQIADATGITKAAVYHH
SRTF1_MUT1 -  MSSTAERIRPGRSGILAAATRLFATHGVSGTSLQQIADATGITKAAVYHH
                       10        20        30        40        50

SRTF1 -       FPTKEEVVVAVLAPALEAIQGIVRTAGAHEDPRAATEAAIIGLADQAVTH
SRTF1_MUT1 -  FPTKEEVVVAVLAPVLEAIHGIVRTAGAHEDPRAATEAAIIGLADQAVTH
                       60        70        80        90       100

SRTF1 -       RQRWAVLLQDAAVEEYVRNNPDHDELFTRLRLLLTGPDPTPGTRLQVSLF
SRTF1_MUT1 -  RQRWAVLLQDAAVEEYVRNNPDHDELFTRLRLLLTGPDPTPGTRLQVSLF
                      110       120       130       140       150

SRTF1 -       LSGLLGPAQDPSCADIDDDALRAGIVRAGRRLLLADDDA
SRTF1_MUT1 -  LSGLLGPAQDPSCADIDDDALRAGIVRAGRRLLLADDDA
                      160       170       180       190
```

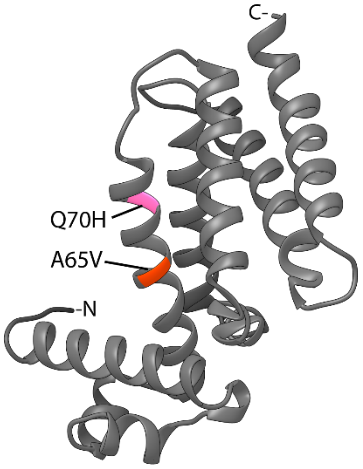

Supplementary Figure 11: Locations of mutations in SRTF1_MUT1 sequence and predicted protein structure for SRTF1. Homology model was built using I-TASSER with KstR chain A from PDB crystal structure 3MNL as a template. Mutations are predicted to lie in the first alpha-helix of the putative ligand binding domain.

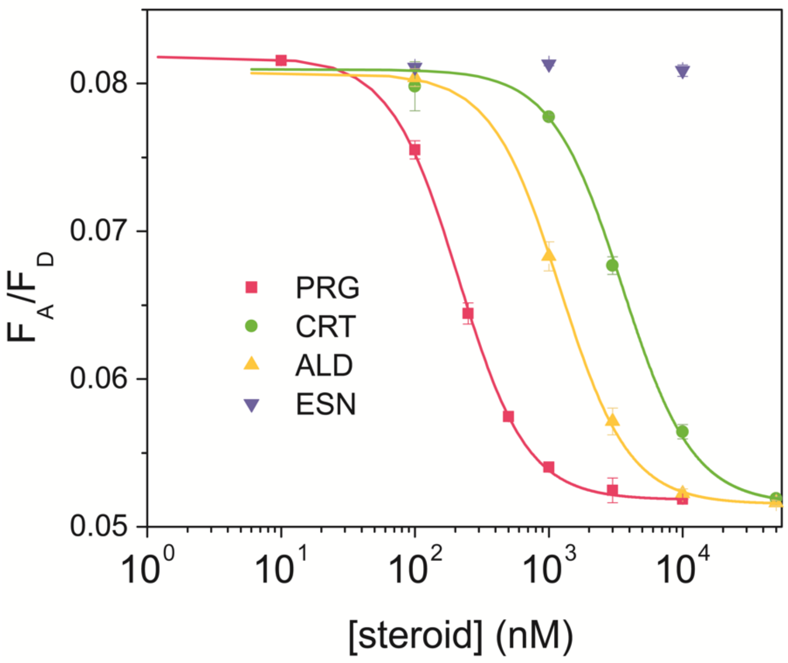

Supplementary Figure 12 Full dose-response curve for Sensor 3 with SRTF1_MUT1 *vs* other sterols (cross-reactivity). Progesterone (PRG): red, Cortisol (CRT): green, Aldosterone (ALD): yellow, Estrone (ESN): blue. Error bars are standard errors over three experiments. Data underlying means are available in the Source Data file.

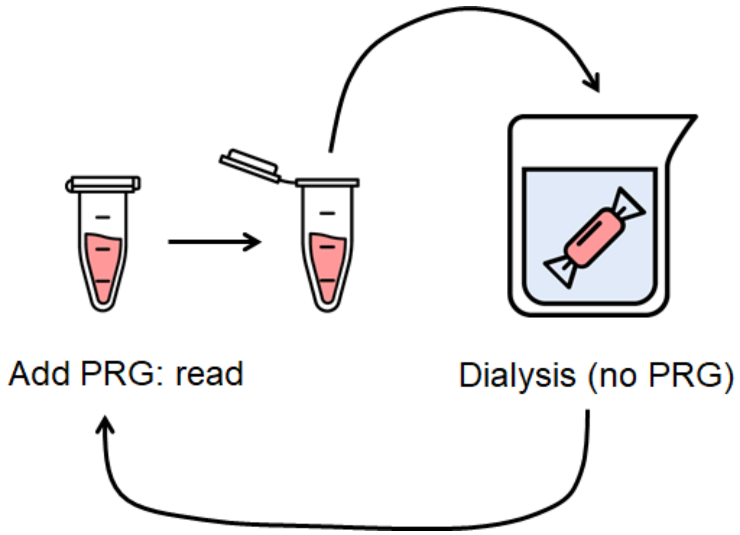

Supplementary Figure 13: Diagram of experiment to assess sensor reversibility. The sensor was assembled and read after a first addition of PRG at 5 different concentrations (0, 25, 75, 100 and 10,000 nM). The sensor was transferred to a dialysis cassette and dialyzed against HEPES overnight to remove PRG. After removal of the PRG, the sensor was transferred back to the plate reader to read the sensor output in the absence of PRG. PRG was added once again at the 5 different concentrations for a second measurement. This cycle was repeated 3 times. Icons (centrifugal tubes- Anthony Ledoux, dialysis bag - Paisley and beaker -mBryan Design) were downloaded free of charge from the Noun Project and were post-colorized.

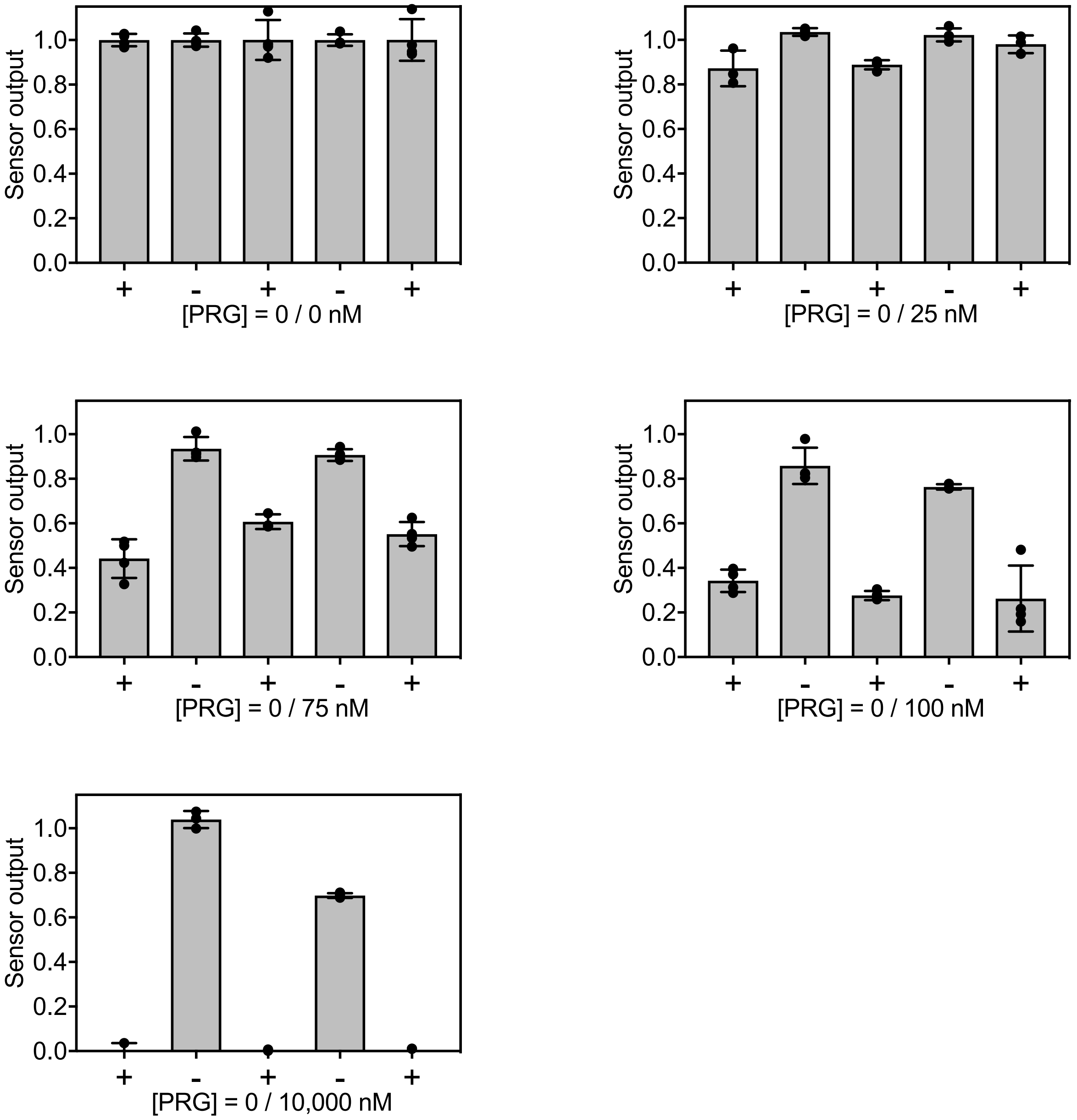

Supplementary Figure 14. Reversibility of the sensor. Evolution of the sensor output after cycles of three adds (+) of PRG to the sensor and two dialysis cycles (-). Each panel corresponds to a specific concentration of PRG added (0, 25, 75, 100 and 10,000nM). With PRG addition, F_A/F_D at [PRG] = 0 nM is normalized to 1, F_A/F_D at [PRG] = 10000 nM is normalized to 0, and linear interpolation is applied for concentrations in between. Without PRG addition, F_A/F_D is scaled, where F_A/F_D at [PRG] = 0 nM is normalized to 1. Error bars are standard errors over more than three experiments. Underlying data are provided within the Source Data file.

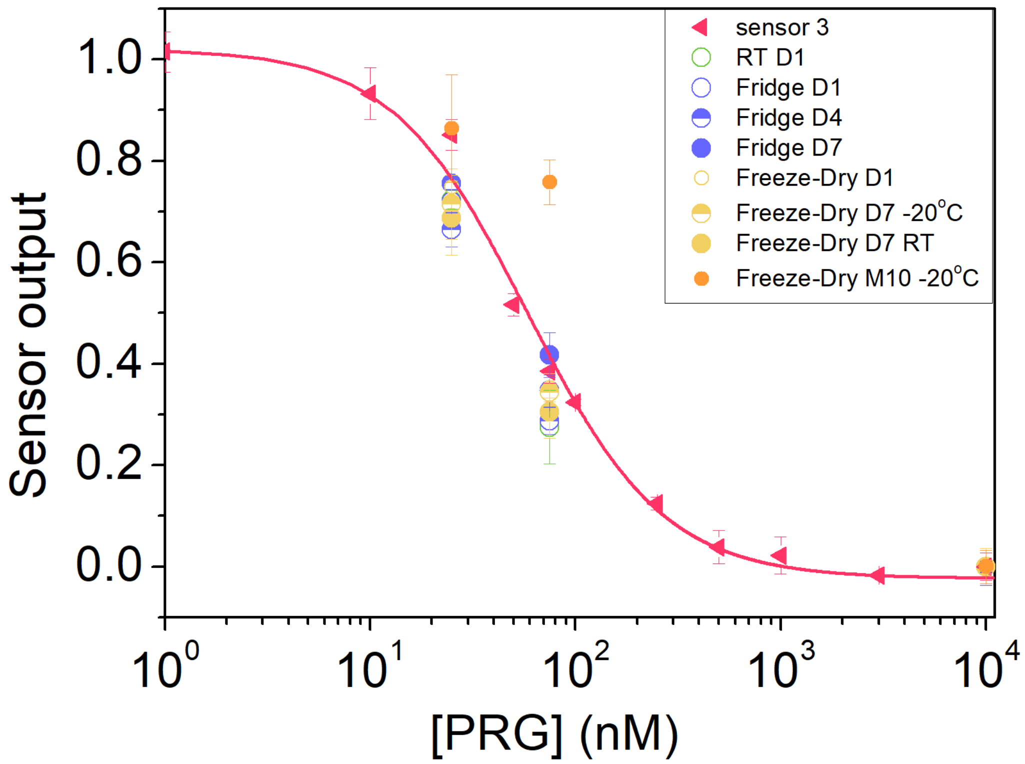

Supplementary Figure 15: Stability of sensor 3 under different storage conditions in the dark: room temperature (RT), in a fridge (4°C), or lyophilized and stored in a freezer (-20ºC) or at RT. D stands for days and M for months. Error bars are standard errors over three experiments. Underlying data are available in the Source Data file.

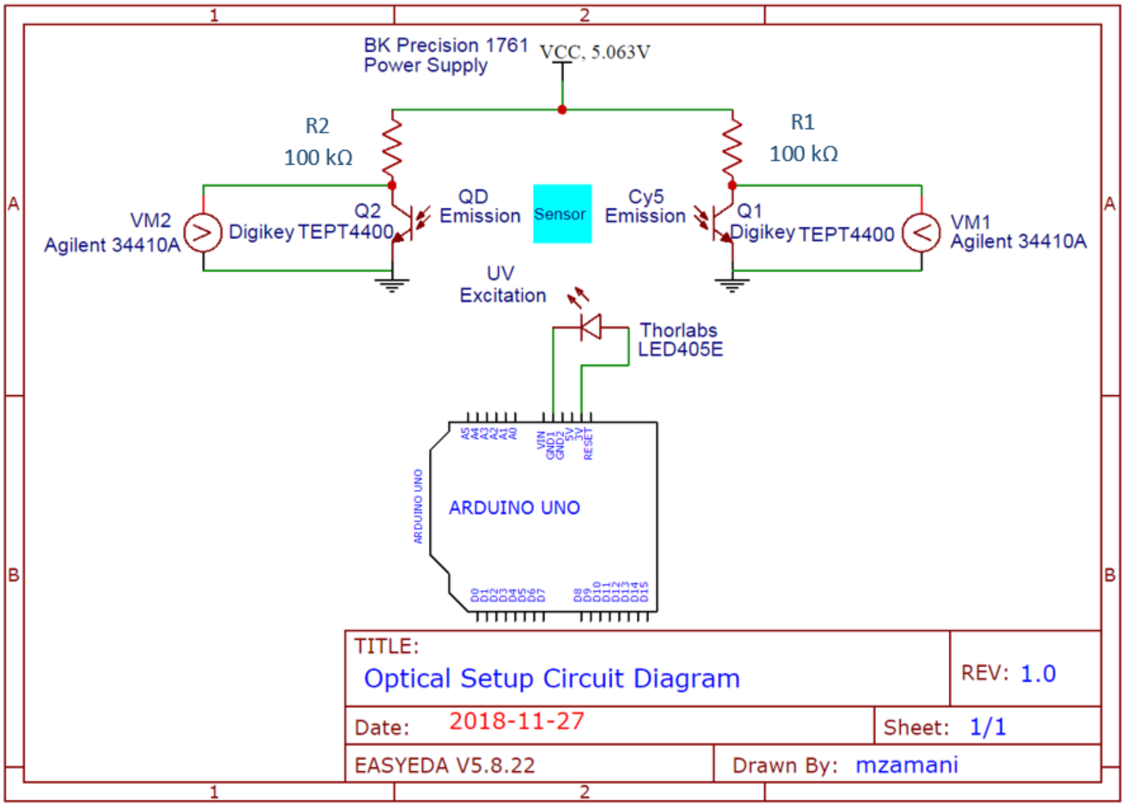

Supplementary Figure 16: Schematic of the phototransistor circuit. An Arduino UNO was used to power the LED used to excite the sample in the cuvette. The QD and Cy5 emissions were detected separately by a phototransistor. Each phototransistor was placed in a common emitter circuit. A voltmeter is used to monitor the change in voltage drop across the phototransistor as light hits the base of the phototransistor. When incident light illuminates the base of the phototransistor, the voltage drop across the phototransistor decreases. A ratio of the amount by which this voltage drop changes in each channel is calculated for samples of the sensor that contain various concentrations of progesterone.

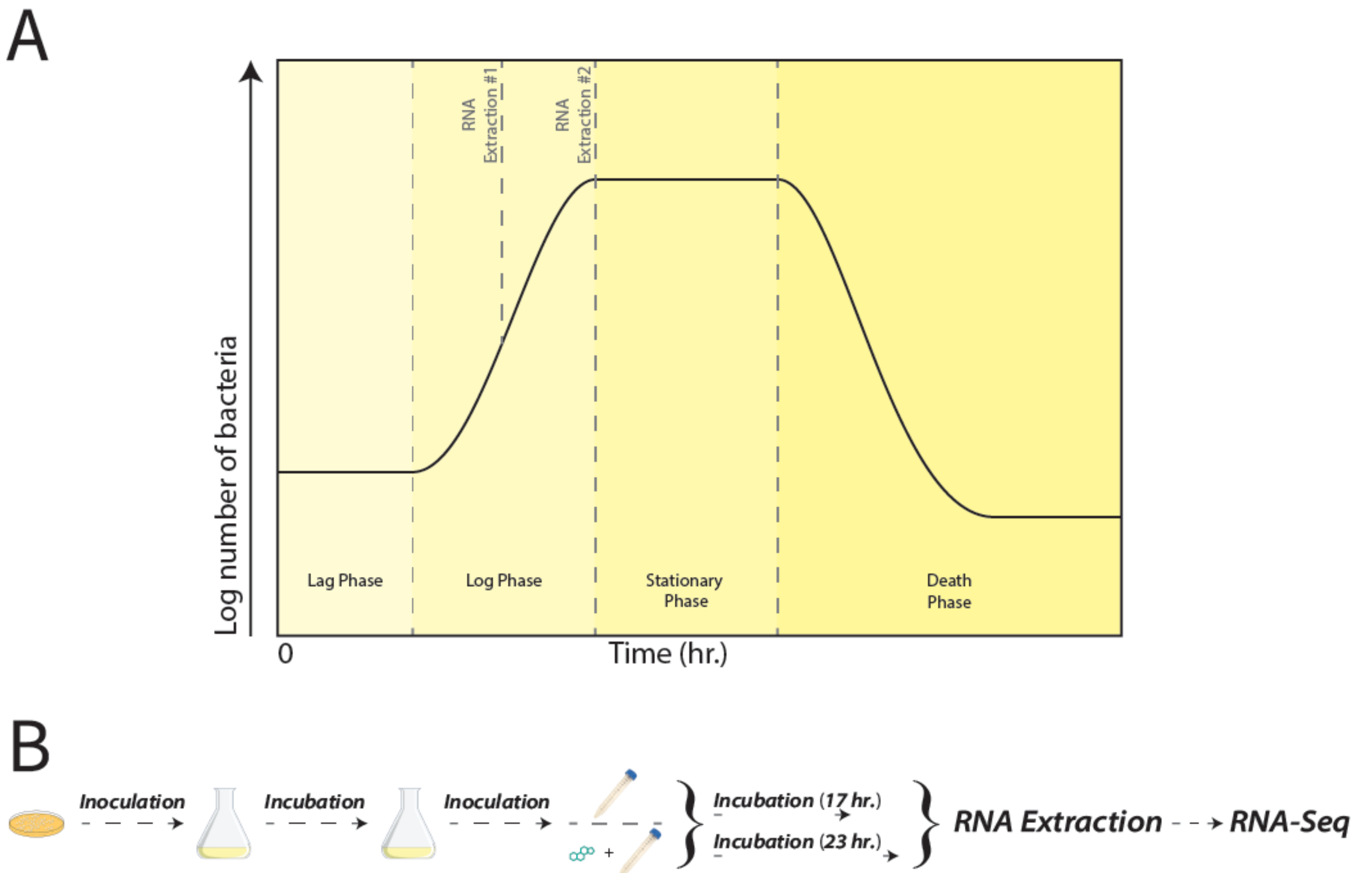

Supplementary Figure 17: Cell growth and extraction protocol for RNA-Seq. A) Typical stages of cell growth over time divided by phases of lag, log, stationary, and death. RNA extractions were performed at mid-log phase and beginning of stationary phase. B) Overview of the culture method for RNA-Seq sample preparation.

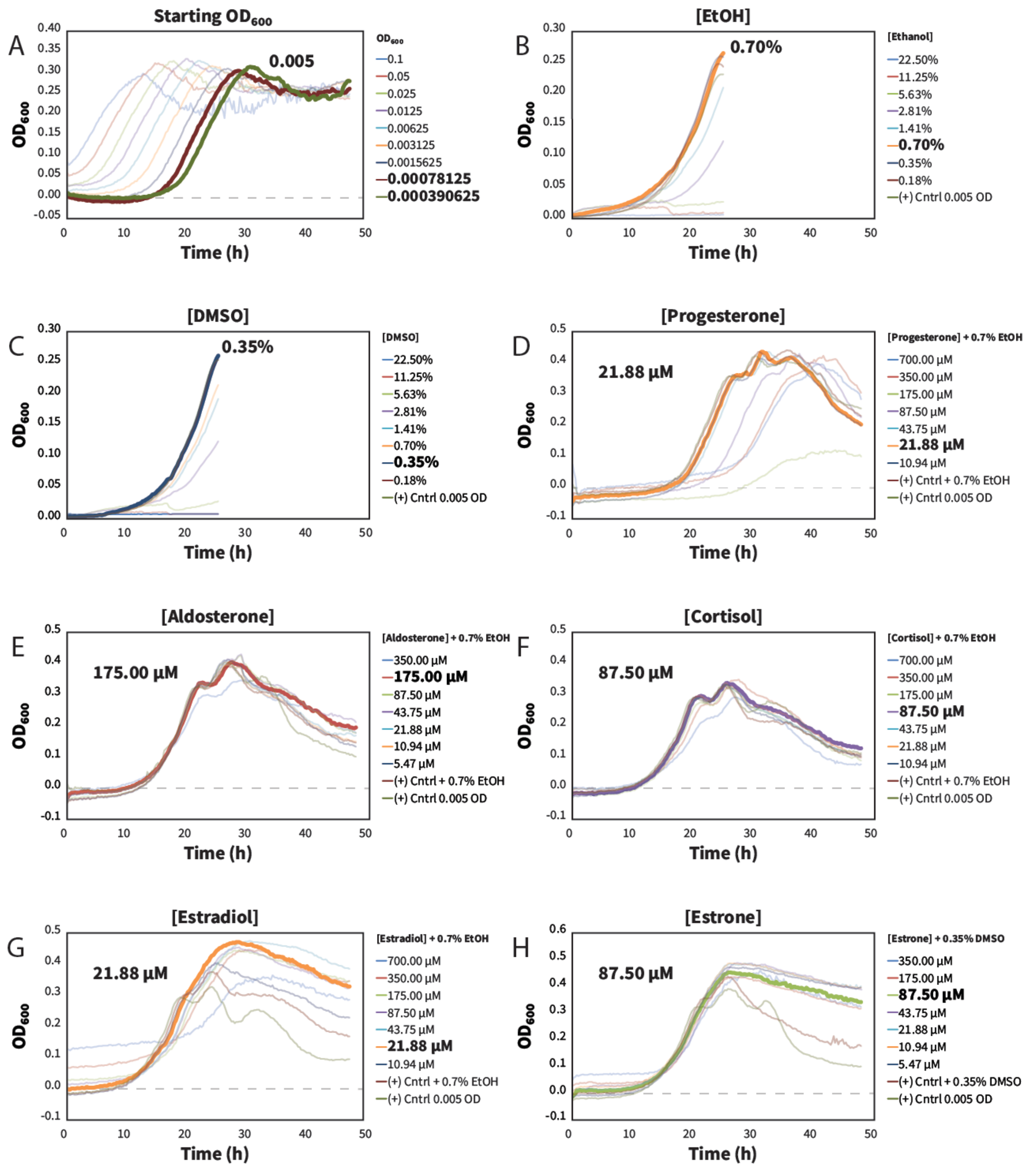

Supplementary Figure 18: Growth curves allowed for the selection of OD, solvent, and steroid concentrations for RNA-Seq. Growth curves done in panels B and C were performed with the M.D. SpectraMax M5 while all other growth curves were done with the Tecan Infinite m200 Pro. **A)** *P. simplex* 6946 OD (1/2) serial dilution growth curve (suggested starting concentration: 0.005 OD). **B)** *P. simplex* 6946 + EtOH (1/2) serial dilution growth curve (suggested starting concentration: 0.7% EtOH). **C)** *P. simplex* 6946 + DMSO (1/2) serial dilution growth curve (suggested starting concentration: 0.35% DMSO). **D)** *P. simplex* 6946 progesterone + EtOH (1/2) serial dilution growth curve (suggested starting concentration: 21.88 µM). **E)** *P. simplex* 6946 aldosterone + EtOH (1/2) serial dilution growth curve (suggested starting concentration: 175 µM). **F)** *P. simplex* 6946 hydrocortisone + EtOH (1/2) serial dilution growth curve (suggested starting concentration: 87.50 µM). **G)** *P. simplex* 6946 estradiol + EtOH (1/2) serial dilution growth curve (suggested starting concentration: 21.88 µM). **H)** *P. simplex* 6946 estrone + DMSO (1/2) serial dilution growth curve (suggested starting concentration: 87.50 µM).

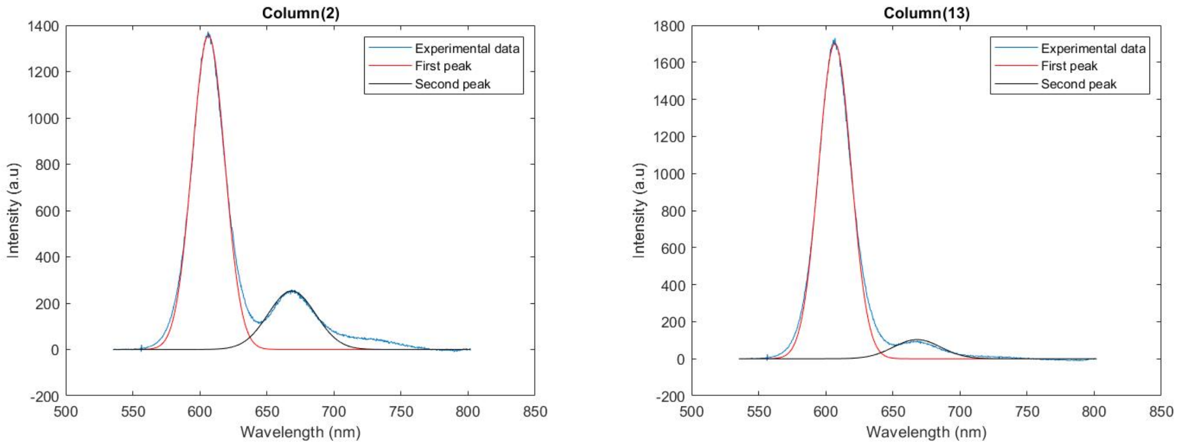

Supplementary Figure 19: Fit and deconvolution of emissions and fluorescent spectra. Total emission spectra were fit to a sum of two Gaussians, one corresponding to QD and the other to Cy5. The integral of each Gaussian was used to calculate the emission for each. Plots shows an example for sensor 1 at two different concentrations, zero and maximum progesterone.

Supplementary Tables

Supplementary Table 1: The SRGI is characterized by an upregulation of genes coding for transcription factors and sterol responsive enzymes when *P. simplex* is induced with progesterone, aldosterone, and hydrocortisone, but not with estradiol and estrone when compared to a solvent control (CNTL).

				Fold Change				
Gene Number	Protein_ID	Locus Tag	Product	PRG *vs* CNTL	ALD *vs* CNTL	CRT *vs* CNTL	ESD *vs* CNTL	ESN *vs* CNTL
5316	AIY19519.1	KR76_27060	Oxidoreductase	5.34	10.22	13.28	1.97	1.54
5317	AIY19520.1	KR76_27065	Hypothetical protein	1.96	2.04	2.10	2.23	0.85
5318	AIY19521.1	KR76_27070	3-ketosteroid-9-alpha-hydroxylase, subunit A	15.98	3.21	7.50	1.32	1.04
5319	AIY20220.2	KR76_27075	Hypothetical protein	8.99	3.13	8.13	0.62	1.90
5320	AIY19522.2	KR76_27080	3-ketosteroid-9-alpha-hydroxylase, subunit B	5.45	2.44	3.79	1.66	0.97
5321	AIY19523.3	KR76_27085	2-hydroxy-6-oxo-6-phenylhexa-2,4-dienoate hydrolase	5.36	1.51	3.10	0.99	0.83
5322	AIY19524.1	KR76_27090	Flavin oxidoreductase	4.16	1.84	3.97	0.76	1.18
5323	AIY19525.1	KR76_27095	2,3-dihydroxybiphenyl 1,2-dioxygenase	17.60	4.44	10.74	1.05	1.00
5324	AIY19526.1	KR76_27100	Hydroxylase	27.08	5.20	12.42	0.98	0.89
5325	AIY20221.1	KR76_27105	Transcriptional regulator, MarR family	1.28	1.83	1.72	1.15	1.01
5326	AIY20222.2	KR76_27110	Transcriptional regulator, IclR family	4.39	2.97	4.66	1.15	0.86
5327	AIY19527.1	KR76_27115	3-ketosteroid-delta1-dehydrogenase	6.77	4.34	6.96	0.38	0.96
5328	AIY19528.1	KR76_27120	Steroid delta-isomerase	7.05	19.39	16.84	-0.87	0.64
5329	AIY19529.1	KR76_27125	3-ketosteroid-delta-1-dehydrogenase	21.77	58.36	47.91	0.53	1.38
5330	AIY20223.2	KR76_27130	Transcriptional regulator, TetR family	2.33	4.40	4.12	0.03	1.13

Supplementary Table 2. Affinity and kinetic measurements associated with aTF binding to DNA in the absence of sterols. Derived from the binding curves in Supplementary Figure 3. *SRTF1o* = oligonucleotide containing the SRTF1 promoter binding site. *SRTF1o* -13,15-T,T = strong binding site with two mutations (see Supplementary Figure 11). Errors of the affinity and kinetic measurements are given by fitting data in Supplementary Figure 3 to a mass transport model using the ForteBio Data Analysis software.

Protein	DNA	K_D (M)	K_D Error (M)	k_{on} ($M^{-1}s^{-1}$)	k_{on} Error ($M^{-1}s^{-1}$)	k_{off} (s^{-1})	k_{off} Error (s^{-1})
SRTF1	*SRTF1o*	4.00E-09	4.11E-10	8.40E+05	6.98E+04	3.36E-03	2.03E-04
	SRTF1o -13,15-T,T	9.19E-06	2.23E-04	2.02E+05	3.45E+06	1.85E+00	3.18E+01
SRTF1-mut1	*SRTF1o*	7.10E-09	3.59E-10	2.34E+05	1.01E+04	1.66E-03	4.33E-05
	SRTF1o -13,15-T,T	1.61E-06	2.53E-04	4.01E+07	4.44E+09	6.46E+01	7.16E+03

Supplementary Table 3: Results of the LOD analysis method for sensors 1, 2 and 3 for PRG, see methods for calculations . IC_{50} and p-value errors are given by the fit of the curve using a Hill function in Origin Pro 8 software, taking into account the 3 measurements of each sensor concentration.

Sensor	IC_{50} nM	p	$Mean_{blank}$	σ_{test}	L_D	LOD nM	LOD 95% CI nM
1	738±27	1.22±0.05	0.27242	0.0017	0.2663	52.6	[49.6 ; 56.7]
2	133±9	1.67±0.15	0.1699	0.0033	0.1591	35.7	[29.1 ; 42.5]
3	57±3	1.57±0.12	0.0996	0.0007	0.0972	15.3	[12.5 ; 17.9]

Supplementary Table 4: Reference ranges for target hormones in serum. All ranges are unions of ranges for adult men and pre-menopausal women. The value for urinary estrogen is for total estrogen.

Hormone	Plasma (nM)	Urine (nM)	Related Compounds	Refs
**Cortisol**	55.18 - 772.5	10.0-138.0	Tetrahydrocortisone<br>Tetrahydrocortisol	1-6
**Testosterone**	0.52 - 37.1	2.0 – 20.0	Epitestosterone	7-11
**Aldosterone**	5.55 - 27.75	N/A	Aldosterone-glucuronide	12,13
**Androstenedione**	0.15 – 10.0	N/A		14,15
**Progesterone**	6.0 – 63.0	3-20	Pregnanediol<br>Pregnanediol-glucuronide	16-22
**Estradiol**<br>**Estrone**	0.037 - 1.29<br>0.026 - 0.74	0.014-0.147	Estrogen-glucuronides<br>Estrogen sulfates	16-21

Supplementary Table 5: Cross-reactivity results of sensor 3 using SRTF1 or SRTF1_MUT1. IC_{50} and p-value errors are given by the fit of the curve using a Hill function in Origin Pro 8 software, taking into account the 3 measurements of each sensor concentration. The cross-reactivity error is calculated using the IC_{50} error.

TF	Steroid	IC_{50} (nM)	P-value	% cross-reactivity
SRTF1	PRG	57±3	1.57±0.12	-
SRTF1	CRT	160±14	1.19±0.10	36±4
SRTF1	ALD	312±42	1.50±0.22	18±3
SRTF1_MUT1	PRG	202±9	1.68±0.11	-
SRTF1_MUT1	CRT	3525±208	1.64±0.14	5.7±0.4
SRTF1_MUT1	ALD	1212±48	1.60±0.11	17±1

Supplementary Table 6. Oligonucleotides labelled with a Cy5 at both 3’ and 5’ end and their complements, used for QD-TF-FRET experiments.

Abb.	Sequence 5’→3’
DNA1-Cy5	5’Cy5-GCCTAACTAGCCGTTCGGCTAGTTATTC-3’Cy5
cDNA1-Cy5	5’-GAATAACTAGCCGAACGGCTAGTTAGGC-3’
DNA2-Cy5	5’Cy5-GCCTAACTAGCCGTTCTGTTAGTTATTC-3’Cy5
cDNA2-Cy5	5'-GAATAACTAACAGAACGGCTAGTTAGGC-3'
Scbd-Cy5	5’Cy5-TGTGCGTGTCCCTCGCTCGGTTTCACGA-3’Cy5
cScbd-Cy5	5’- TCGTGAAACCGAGCGAGGGACACGCACA-3’

Supplementary Table 7. Oligonucleotides used for BLI experiments.

Name	Sequence 5'→3'	Name
BLI-*SRTF1o-F*	5'Biotin-AAATTGTCCCGGGCCTTTCTCCGCGAACTAGCCGTTCGGCTAGTTTACTC-3'	BLI-*SRTF1o-F*
BLI-*SRTF1o-R*	5'-GAGTAAACTAGCCGAACGGCTAGTTCGCGGAGAAAGGCCCGGGACAATTT-3'	BLI-*SRTF1o-R*
BLI-*SRTF1o*-13,15-T,T-F	5'Biotin-AAATTGTCCCGGGCCTTTCTCCGCGAACTAGCCGTTCTGTTAGTTTACTC-3'	BLI-*SRTF1o*-13,15-T,T-F
BLI-*SRTF1o*-13,15-T,T-R	5'-GAGTAAACTAACAGAACGGCTAGTTCGCGGAGAAAGGCCCGGGACAATTT-3'	BLI-*SRTF1o*-13,15-T,T-R
BLI-SCRM-F	5'Biotin-AAATTGTCCCGGGCCTTTCTCCGCGCGTCTGGTAACCCTTAGGATTACTC-3'	BLI-SCRM-F
BLI-SCRM-R	5'-GAGTAATCCTAAGGGTTACCAGACGCGCGGAGAAAGGCCCGGGACAATTT-3'	BLI-SCRM-R

Supplementary Table 8. Primers used in this study

Name	Sequence
pJ02dsrtf1o_F	ttgaagacttttgacagctagctcagtcctaggtactgtgctagcaactagccgttcggctagttaaagtcttcaa
pJ02dsrtf1o_R	ttgaagactttaactagccgaacggctagttgctagcacagtacctaggactgagctagctgtcaaaagtcttcaa
SRTF1-6HIS_CD_F	ttgaagacttaatgtcgtcaacggctgaacgcatccgtcctg
SRTF1-6HIS_CD_R	ttgaagacttacctttagtgatggtggtgatgatgtgacccggcgtcgtcgtccgcaagtaataaacgacgtccg
BsaI_removal_R1	ttgaagacttctgcagcggccgctactagtagggctctagcgtataaacg
BsaI_removal_F1	ttgaagacttcgcttctagagactagtggggctcaggagttgac
BsaI_removal_F2	ttgaagacttgcaggcttcctcgctcactgactc
BsaI_removal_R2	ttgaagacttagcggccgcgaattccagaaatc
pJ02d-DEVECT_BsaI site_v2_F	ttgaagaccgggtcacatcatcaccaccatcactaaagg
pJ02d-DEVECT_BsaI_site_v2_R	ttgaagacttctacaacttcttcctttgtagggaaatggtgatataccgcc
stuffer_insert_F	tctcgaagacttgtagtgagacctgccacctgaggtctaagaaaccgaattcattattatattaacctattaggggtctc aggtcaagtcttcgaga
suffer_insert_R	tctcgaagacttgacctgagacccctaataggttaatataataatgaattcggtttcttagacctcaggtggcaggtctc actacaagtcttcgaga
SRTF1_vardomain_epPCR_F	ttggtctctgtagtggccgtgttagcacct
SRTF1_vardomain_epPCR_R	ttggtctccgacccggcgtcgtcgtcc

Supplementary Information References

1 Invitti, C., Pecori Giraldi, F., de Martin, M. & Cavagnini, F. Diagnosis and management of Cushing's syndrome: results of an Italian multicentre study. Study Group of the Italian Society of Endocrinology on the Pathophysiology of the Hypothalamic-Pituitary-Adrenal Axis. *J Clin Endocrinol Metab* **84**, 440-448, doi:10.1210/jcem.84.2.5465 (1999).

2 Dickstein, G. *et al.* Adrenocorticotropin stimulation test: effects of basal cortisol level, time of day, and suggested new sensitive low dose test. *J Clin Endocrinol Metab* **72**, 773-778, doi:10.1210/jcem-72-4-773 (1991).

3 Nieman, L. K. *et al.* The diagnosis of Cushing's syndrome: an Endocrine Society Clinical Practice Guideline. *J Clin Endocrinol Metab* **93**, 1526-1540, doi:10.1210/jc.2008-0125 (2008).

4 Wood, P. J., Barth, J. H., Freedman, D. B., Perry, L. & Sheridan, B. Evidence for the low dose dexamethasone suppression test to screen for Cushing's syndrome--recommendations for a protocol for biochemistry laboratories. *Ann Clin Biochem* **34 (Pt 3)**, 222-229, doi:10.1177/000456329703400302 (1997).

5 Blethen, S. L. & Chasalow, F. I. Overnight dexamethasone suppression test: normal responses and the diagnosis of Cushing's syndrome. *Steroids* **54**, 185-193 (1989).

6 Crowley, S., Hindmarsh, P. C., Holownia, P., Honour, J. W. & Brook, C. G. D. The Use of Low-Doses of Acth in the Investigation of Adrenal-Function in Man. *Journal of Endocrinology* **130**, 475-479, doi:DOI 10.1677/joe.0.1300475 (1991).

7 Morley, J. E. & Perry, H. M., 3rd. Androgen deficiency in aging men: role of testosterone replacement therapy. *J Lab Clin Med* **135**, 370-378, doi:10.1067/mlc.2000.106455 (2000).

8 Manni, A. *et al.* Bioavailability of albumin-bound testosterone. *J Clin Endocrinol Metab* **61**, 705-710, doi:10.1210/jcem-61-4-705 (1985).

9 New, M. I. & Josso, N. Disorders of gonadal differentiation and congenital adrenal hyperplasia. *Endocrinol Metab Clin North Am* **17**, 339-366 (1988).

10 Dumesic, D. Hyperandrogenic anovulation: a new view of polycystic ovary syndrome. *Postgrad Obstet Gynecol* **15**, 1-6 (1995).

11 Moon, J. Y. *et al.* Reference ranges for urinary levels of testosterone and epitestosterone, which may reveal gonadal function, in a Korean male population. *J Steroid Biochem Mol Biol* **140**, 100-105, doi:10.1016/j.jsbmb.2013.12.001 (2014).

12 Hurwitz, S., Cohen, R. J. & Williams, G. H. Diurnal variation of aldosterone and plasma renin activity: timing relation to melatonin and cortisol and consistency after prolonged bed rest. *J Appl Physiol (1985)* **96**, 1406-1414, doi:10.1152/japplphysiol.00611.2003 (2004).

13 Williams, G. H., Cain, J. P., Dluhy, R. G. & Underwood, R. H. Studies of the control of plasma aldosterone concentration in normal man. I. Response to posture, acute and chronic volume depletion, and sodium loading. *J Clin Invest* **51**, 1731-1742, doi:10.1172/JCI106974 (1972).

14 Elias, A. N., Pandian, M. R. & Rojas, F. J. Serum levels of androstenedione, testosterone and dehydroepiandrosterone sulfate in patients with premature ovarian failure to age-matched menstruating controls. *Gynecol Obstet Invest* **43**, 47-48 (1997).

15 Buttler, R. M. *et al.* Comparison of eight routine unpublished LC-MS/MS methods for the simultaneous measurement of testosterone and androstenedione in serum. *Clinica chimica acta; international journal of clinical chemistry* **454**, 112-118, doi:10.1016/j.cca.2016.01.002 (2016).

16 Traggiai, C. & Stanhope, R. Delayed puberty. *Best Pract Res Clin Endocrinol Metab* **16**, 139-151, doi:10.1053/beem.2001.0186 (2002).

17 Iughetti, L. *et al.* Diagnosis of central precocious puberty: endocrine assessment. *J Pediatr Endocrinol Metab* **13 Suppl 1**, 709-715 (2000).

18 Kligman, I. & Rosenwaks, Z. Differentiating clinical profiles: predicting good responders, poor responders, and hyperresponders. *Fertil Steril* **76**, 1185-1190 (2001).

19 Ismail, A. A. & Barth, J. H. Endocrinology of gynaecomastia. *Ann Clin Biochem* **38**, 596-607, doi:10.1258/0004563011900993 (2001).

20 Cummings, S. R. *et al.* Endogenous hormones and the risk of hip and vertebral fractures among older women. *New England Journal of Medicine* **339**, 733-738, doi:Doi 10.1056/Nejm199809103391104 (1998).

21 Elmlinger, M. W., Kuhnel, W. & Ranke, M. B. Reference ranges for serum concentrations of lutropin (LH), follitropin (FSH), estradiol (E2), prolactin, progesterone, sex hormone-binding globulin (SHBG), dehydroepiandrosterone sulfate (DHEAS), cortisol and ferritin in neonates, children and young adults. *Clin Chem Lab Med* **40**, 1151-1160, doi:10.1515/CCLM.2002.202 (2002).

22 Stanczyk, F. Z. *et al.* Urinary progesterone and pregnanediol. Use for monitoring progesterone treatment. *J Reprod Med* **42**, 216-222 (1997).
