# Supplementary material for: A progesterone biosensor derived from microbial screening
Source: Nat Commun. 2020 Mar 9;11:1276. doi: 10.1038/s41467-020-14942-5 (PMC7062782; doi:10.1038/s41467-020-14942-5)
Supplement: Supplementary file 2 — Reporting Summary [file 41467_2020_14942_MOESM2_ESM.pdf]

nature research

Corresponding author(s): James Galagan

Last updated by author(s): Jan 17, 2020

Reporting Summary

Nature Research wishes to improve the reproducibility of the work that we publish. This form provides structure for consistency and transparency in reporting. For further information on Nature Research policies, see Authors & Referees and the Editorial Policy Checklist .

Statistics

For all statistical analyses, confirm that the following items are present in the figure legend, table legend, main text, or Methods section.

n/a	Confirmed	
[ ]	[x]	The exact sample size (*n*) for each experimental group/condition, given as a discrete number and unit of measurement
[ ]	[x]	A statement on whether measurements were taken from distinct samples or whether the same sample was measured repeatedly
[ ]	[x]	The statistical test(s) used AND whether they are one- or two-sided<br>*Only common tests should be described solely by name; describe more complex techniques in the Methods section.*
[x]	[ ]	A description of all covariates tested
[x]	[ ]	A description of any assumptions or corrections, such as tests of normality and adjustment for multiple comparisons
[x]	[ ]	A full description of the statistical parameters including central tendency (e.g. means) or other basic estimates (e.g. regression coefficient) AND variation (e.g. standard deviation) or associated estimates of uncertainty (e.g. confidence intervals)
[x]	[ ]	For null hypothesis testing, the test statistic (e.g. *F*, *t*, *r*) with confidence intervals, effect sizes, degrees of freedom and *P* value noted<br>*Give P values as exact values whenever suitable.*
[x]	[ ]	For Bayesian analysis, information on the choice of priors and Markov chain Monte Carlo settings
[x]	[ ]	For hierarchical and complex designs, identification of the appropriate level for tests and full reporting of outcomes
[x]	[ ]	Estimates of effect sizes (e.g. Cohen's *d*, Pearson's *r*), indicating how they were calculated

Our web collection on statistics for biologists contains articles on many of the points above.

Software and code

Policy information about availability of computer code

Data collection	Standard software installed on the instruments described in the Methods was used for data collection (e.g. spectroscopy, fluorescence measurements, bioLayer interferometry). LabView was used for data collection for the low-cost electronic reader.
Data analysis	For sequence alignment, adaptor sequences were removed from reads and low quality bases trimmed from both ends using Cutadapt. Reads were aligned to the reference genome with Bowtie. BAM files were sorted and indexed using Samtools.<br><br>For RNA-Seq analysis from aligned sequences, transcript assembly and expression quantification was performed using Cufflinks. All resulting raw expression counts were normalized as a group using deseq. A custom matlab script was then used to calculate fold changes of normalized counts for each gene between each sterol exposure experiment and its corresponding vehicle control (this custom matlab script can be made available on request).<br><br>Origin Pro Software and the R package ggplot2 was used for data analysis and graphing as indicated in the Methods.

For manuscripts utilizing custom algorithms or software that are central to the research but not yet described in published literature, software must be made available to editors/reviewers. We strongly encourage code deposition in a community repository (e.g. GitHub). See the Nature Research guidelines for submitting code & software for further information.

Data

Policy information about availability of data

All manuscripts must include a data availability statement. This statement should provide the following information, where applicable:

- Accession codes, unique identifiers, or web links for publicly available datasets
- A list of figures that have associated raw data
- A description of any restrictions on data availability

All RNA-Seq data that support this paper will be deposited in the NCBI Sequence Read Archive prior to publication. All iv-ChIP-seq data that support this paper have been submitted to the Gene Expression Omnibus with the accession number GSE131041. Source data from which figures 2B, 2C, 3C, 3E, 3F, 3G, 3H, 4B, and Supplementary figures 5, 9, 10, 12, 14, and 15 are generated are located in the Source Data file. Other relevant data, custom software, and analysis scripts will be made available on request

Field-specific reporting

Please select the one below that is the best fit for your research. If you are not sure, read the appropriate sections before making your selection.

- [x] Life sciences
- [ ] Behavioural & social sciences
- [ ] Ecological, evolutionary & environmental sciences

For a reference copy of the document with all sections, see nature.com/documents/nr-reporting-summary-flat.pdf

Life sciences study design

All studies must disclose on these points even when the disclosure is negative.

Sample size	Samples sizes for each measurement are described in the text. A minimum of 3 replicates for each measurement was performed. The primary purpose of replicates was to define measurement errors, rather than to achieve statistical power.
Data exclusions	No data were excluded from replicate measurements.
Replication	N/A
Randomization	N/A
Blinding	N/A

Reporting for specific materials, systems and methods

We require information from authors about some types of materials, experimental systems and methods used in many studies. Here, indicate whether each material, system or method listed is relevant to your study. If you are not sure if a list item applies to your research, read the appropriate section before selecting a response.

Materials & experimental systems

n/a	Involved in the study	
[ ]	[x]	Antibodies
[x]	[ ]	Eukaryotic cell lines
[x]	[ ]	Palaeontology
[x]	[ ]	Animals and other organisms
[x]	[ ]	Human research participants
[x]	[ ]	Clinical data

Methods

n/a	Involved in the study	
[ ]	[x]	ChIP-seq
[x]	[ ]	Flow cytometry
[x]	[ ]	MRI-based neuroimaging

Antibodies

Antibodies used	6x-His Tag Monoclonal Antibody His.H8 from Thermofisher Scientific.
Validation	This antibody is designed to recognize the 6x-His Tag and has been validated by both the manufacturer and through the routine use of this antibody in the Galagan lab to pull-down His-tagged proteins through immunopurification.

ChIP-seq

Data deposition

☒ Confirm that both raw and final processed data have been deposited in a public database such as GEO.

☒ Confirm that you have deposited or provided access to graph files (e.g. BED files) for the called peaks.

Data access links<br>*May remain private before publication.*	The data are available in GEO as GSE131041. The data a private pending submission, but can be viewed by reviewers at the following link: https://www.ncbi.nlm.nih.gov/geo/query/acc.cgi?acc=GSE131041 using the following token: mhmlmyoevhorfuf
Files in database submission	AIY_peaks.xlsx<br>GEO_submission_biosensor.xls<br>JG0331_Psim_AIY_1_R1.fastq.gz<br>JG0331_Psim_AIY_1.sorted.bam<br>JG0331_Psim_AIY_2_R1.fastq.gz<br>JG0331_Psim_AIY_2.sorted.bam<br>JG0331_Psim_WM_1-2_R1.fastq.gz<br>JG0331_Psim_WM_1-2.sorted.bam<br>JG0331_Psim_WM_1_R1.fastq.gz<br>JG0331_Psim_WM_1.sorted.bam
Genome browser session<br>(e.g. UCSC)	The reference genome we used is not available through UCSC

Methodology

Replicates	The data were generated using a novel in vitro ChIP-Seq methodology as described in the manuscript. Two replicates of in vitro ChIP-seq and two replicates of antibody-free mock-ChIP controls were performed.
Sequencing depth	Total Reads -<br>ChIP 1: 9129131<br>ChIP 2: 10656783<br><br>Mock 1: 8745532<br>Mock 2: 40322724
Antibodies	Anti-his tag HIS.H8 (Thermofisher)
Peak calling parameters	As we were interested primarily with identifying potential binding sequences within the SRGI that we would subsequently validate, we identified strong peaks by eye within this region. Three peaks were identified within this region all of which included a forward/reverse peak shift. The strongest binding site had a fold enrichment of 70.9 over the mean coverage and we independently experimentally verified this site as described in the text.
Data quality	Data quality was ensured through the use of fastqc to ensure read quality, percent passing reads aligned to ensure acceptable alignments, sequencing depth as described above to ensure ample read coverage, and replicate experiments.
Software	Illumina adapter sequences were removed with Cutadapt, and reads were aligned using Bowtie2.
